# Cluster-Engineered Titanium Metal−Organic Aerogels as Tunable Platforms for Post-synthetic Doping and Enhanced Photocatalytic Hydrogen Production

**DOI:** 10.1021/acs.inorgchem.5c03886

**Published:** 2025-12-15

**Authors:** Naia Luengo, Ane Ciruela-Zunzunegui, Maite Perfecto-Irigaray, Oscar Castillo, Pilar Ferrer, Matthijs A. van Spronsen, Sonia Pérez-Yáñez, Garikoitz Beobide

**Affiliations:** † Department of Organic and Inorganic Chemistry, Faculty of Science and Technology, 16402 University of the Basque Country (UPV/EHU), 48940 Leioa, Spain; ‡ Department of Chemistry, 9304 Università degli Studi di Milano, Via Golgi 19, 20133 Milan, Italy; § ISIS Neutron and Muon Source, STFC Rutherford Appleton Laboratory, Didcot OX11 0QX, U.K.; ∥ BCMaterials, Basque Center for Materials, Applications and Nanostructures, UPV/EHU Science Park, Leioa 48940, Spain; ⊥ Diamond Light Source, Harwell Science and Innovation Campus, Didcot OX11 0DE, U.K.

## Abstract

Metal−organic gels (MOGs) and their derived aerogels (MOAs) offer an alternative to crystalline MOFs, combining the coordination-driven tunability with the flexibility, hierarchical porosity, and easy processability of sol–gel polymers. Their noncrystalline nature enables the integration of functional units without crystallization constraints, facilitating diverse uses, and drawing recent attention for photocatalytic applications. Herein we report the design of a new approach to prepare a titanium-based MOA synthesized via a two-step strategy involving a preformed titanium oxo-cluster ([Ti_8_O_8_(benzoato)_16_]), and a subsequent ligand exchange with benzene-1,3,5-tricarboxylato ligands. A combined chemical, microstructural, and NEXAFS analysis confirms the retention of Ti_8_ cluster and the presence of uncoordinated −COOH groups after meso-macroporous gel formation. Those enabled a subsequent homogeneous incorporation of single-atom site co-catalysts via coordination with Ru, Co, Ni, and Cu complexes bearing terpyridine, bipyridine, and phenanthroline N-ligands. Photocatalytic hydrogen evolution under 365 nm LED irradiation exhibited significant activity (110 μmol·g^–1^·h^–1^), which further increased upon functionalization. The MOAs functionalized with Ru- and Cu-terpyridine complexes showed the highest performance (167 and 164 μmol·g^–1^·h^–1^, respectively), surpassing even Pt-loaded analogues and highlighting the role of terpyridine in facilitating multielectron storage. The system also showed stable long-term performance up to 24 h.

## Graphical abstract

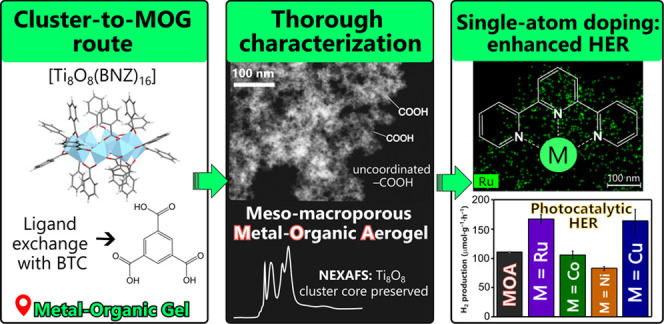

## 1 Introduction

Metal−organic frameworks (MOFs) have emerged as a widely studied family of porous materials due to their high surface-area-to-volume ratios and modular architectures combining organic and inorganic components.^1,2^ Their versatility has enabled a wide range of applications, including gas separation and storage, sensing, catalysis, drug delivery, and others.^3–5^ Among these, photocatalysis has become one of the most active areas of research, particularly in relation to solar-driven hydrogen production and CO_2_ reduction.^6,7^ Current environmental concerns and the increasing demand for sustainable energy alternatives further underscore the relevance of this field.^8,9^

The photocatalytic mechanism in this class of metal−organic assemblies typically involves the excitation of an electron from the highest occupied molecular orbital (HOMO), which is primarily composed of π-type orbitals of the ligand, to the lowest unoccupied molecular orbital (LUMO), consisting of vacant d-orbitals of the metal,^10^ following the so-called ligand-to-metal charge transfer (LMCT) mechanism upon photoexcitation.^11^ These processes help to suppress recombination and extend charge carrier lifetimes and thus recent advances have focused on improving LMCT pathways and spatial charge separation through targeted design strategies.^12^ However, despite their crystalline precision, MOFs often present limitations in mass transport and cofunctionalization during synthesis, which can restrict their catalytic potential.^13^

To address these challenges, metal−organic gels (MOGs) and their aerogels (MOAs) have afterward been developed as related materials with similar coordination chemistry but very different structural and textural properties.^14–16^ These materials are composed of meso- and macroporous networks formed by the assembling of metal−organic nanoparticles, and therefore lacking of long-range crystalline ordering in most of the cases. As a consequence, their extrinsic porosity and larger pore sizes facilitate faster diffusion of reactants and products, improving performance in catalytic processes.^16^ Additionally, the rapid and less selective polymerization governing MOG synthesis facilitates coligand incorporation and defect formation, thereby offering great opportunities for the design of materials with new functionalities.^17,18^ All these features have motivated extensive research demonstrating the great potential of MOGs as multifunctional porous platforms.^19^ Ongoing efforts continue to explore MOGs as high-performance, scalable alternatives for environmental and catalytic applications.^20,21^

Among this class of materials, we recently developed MOGs composed of titanium oxo-clusters cross-linked by benzene-1,4-dicarboxylato (BDC) and/or 2-aminobenzene-1,4-dicarboxylato (aBDC) ligands (acting as bis-bidentate bridging linkers), which stand out in the photocatalytic CO_2_ reduction to alcohols.^17,18^ The performance of these MOGs results in methanol production rates that are 5 to 10 times higher than those of analogous microcrystalline MOFs, such as MIL-125 and MIL-125-NH_2_. This same MOG family has also demonstrated the ability to photocatalyse the hydrogen evolution reaction (HER), that is significantly enhanced when platinum is used as co-catalyst.^22^

Herein, we describe the synthesis, characterization, doping process and photocatalytic HER performance of a new series of Ti-based MOGs. In a departure from single-step MOG syntheses of systems mentioned above, to enhance the control over the final structure, in this case we employed a two-step approach involving the presynthesis of a discrete octanuclear titanium cluster that is afterward cross-linked by benzene-1,3,5-tricarboxylato (BTC) ligands in a second step to yield a meso-macroporous MOA (denoted as Ti_8_BTC). Specifically, it builds upon the structural motifs and preformed cluster approach found in MIP-207, a crystalline microporous MOF in which octanuclear titanium-oxo clusters and BTC linkers assemble into a two-dimensional coordination framework with the formula [Ti_8_(μ-O)_8_​(μ-acetato-κ*O*:κ*O*′)_8_​(μ_4_-BTC-κ*O*:κ*O*′:​κ*O*″:κ*O*‴)_4_]_*n*_.^23^ In this framework, BTC coordinates through only two of its three carboxylate groups, leaving the third group free and protonated (−COOH), in contrast to the BDC linker in MIL-125, which fully coordinates through both of its carboxylate groups. Accordingly, the cross-linking of preformed octanuclear titanium units with BTC during the Ti_8_BTC formation produces free −COOH groups at the pore surface that offer suitable sites to incorporate different co-catalyst species by post-synthetic modifications to explore alternatives for the use of platinum in the photocatalytic HER. Therefore, the influence of the post-synthetic single-atom doping process using Ru, Co, Ni, and Cu complexes on the light absorption properties and HER performance under UV light illumination is analyzed. This work aims to bridge the gap between the inherent advantages of titanium-oxo clusters and the tunable properties of MOGs, providing insights into the design of efficient photocatalytic materials for hydrogen production, with potential implications for other energy-related processes such as CO_2_ reduction or water splitting, a critical step toward sustainable energy solutions.

## 2 Experimental Section

### 2.1 Synthesis

Titanium­(IV) *n*-butoxide (TNBT, 98%, 1.00 g·mL^–1^ at 20 °C), benzoic acid (HBNZ, 99.5%) benzene-1,3,5-tricarboxylic acid (H_3_BTC, 99%), 1,10-phenanthroline (PHEN, 99%), ruthenium­(III) chloride trihydrate (RuCl_3_·3H_2_O, 99%), nickel­(II) chloride hexahydrate (NiCl_2_·6H_2_O, 99%), cobalt­(II) chloride hexahydrate (CoCl_2_·6H_2_O, 99%), and copper­(II) chloride (CuCl_2_, 99%) were purchased from Sigma-Aldrich. Absolute ethanol (EtOH), hydrochloric acid (HCl, 37%), *N*,*N*-dimethylformamide (DMF, 99%), and butan-2-ol (2-BuOH, 98%) from labKem were also used in this work. 2,2′:6′,2″-terpyridine (TPY, 99%) was purchased from Fluorochem, 2,2′-bipyridine (BPY, 98%) from Fluka, and acetonitrile (AcCN, 99.9%) from Scharlau.

#### 2.1.1 Preamble

The synthesis of the MOGs was based on an experimental procedure previously described for similar metal−organic systems.^22^ In that publication, butan-2-ol (2-BuOH) and *N*,*N*-dimethylformamide (DMF) were used as synthesis solvents to react titanium­(IV) *n*-butoxide with benzene-1,4-dicarboxylic acid in the presence of hydrochloric acid at 80 °C. This reaction involves the formation of the titanium oxo-cluster and the growth of the coordination polymer in a single synthesis step. However, in the present study, a two-step synthesis process was proposed using benzene-1,3,5-tricarboxylate (BTC) instead of benzene-1,4-dicarboxylate as linker. The first stage involved the preparation of an octanuclear titanium cluster with formula [Ti_8_(μ-O)_8_(μ-BNZ)_16_]·​(CH_3_CN)_2_·H_2_O (BNZ: benzoato) which was used in a second stage as a preformed metal node in a polymerization process driven by the ligand exchange reaction with BTC. Another significant difference is that the polymerization process leading to the formation of the MOG was carried out in the absence of hydrochloric acid and at room temperature, in an attempt to preserve the structural integrity of the preformed octanuclear cluster.

#### 2.1.2 Synthesis of the Precursor [Ti_8_(μ-O)_8_​(μ-BNZ)_16_]·​(CH_3_CN)_2_·​H_2_O (Ti_8_BNZ)

Following the conditions described in the literature,^24^ benzoic acid (2.84 g, 23.14 mmol) was dissolved in dry acetonitrile (25 mL) by heating at 60 °C for 10 min in a closed vessel. Simultaneously, a second solution was prepared by adding titanium­(IV) *n*-butoxide (785 μL, 2.26 mmol) in dry acetonitrile (5 mL) and stirred for 10 min in a closed vessel. After this time, the benzoic acid solution was added rapidly in a single addition to the titanium­(IV) *n*-butoxide solution. The resulting mixture was stirred for 5 min at room temperature and then subjected to solvothermal conditions in an autoclave reactor, which was heated in an oven at 120 °C for 17 h. At the end of the reaction, the mixture was allowed to cool down to room temperature and colorless needle-shaped Ti_8_BNZ crystals were obtained at the bottom of the vessel. The product was thoroughly washed with acetonitrile and filtered under vacuum.

#### 2.1.3 Synthesis of the Ti_8_BTC MOG

To synthesize Ti_8_BTC MOG, benzene-1,3,5-tricarboxylic acid (0.70 g, 3.30 mmol) was first dissolved in 2-BuOH (14 mL) by heating to 60 °C. In parallel, a solution of the octanuclear precursor (Ti_8_BNZ: 1.01 g, 0.40 mmol) was prepared in a mixture of 2-BuOH and DMF (2 and 10 mL, respectively). The H_3_BTC solution was allowed to cool down to room temperature before being added under continuous stirring to the solution containing the metal. After addition, the reaction vessel was closed to minimize the absorption of atmospheric moisture. The Ti_8_BTC metal−organic gel was formed after 15 min and it was left to age for 24 h before processing.

Thereafter, to remove unreacted species, the gels underwent successive solvent exchanges, initially with a butan-2-ol/DMF (2:1) mixture, followed by a butan-2-ol/DMF/ethanol (1:1:1) mixture, and finally with absolute ethanol to replace residual butan-2-ol and DMF.

#### 2.1.4 Post-synthetic Metalation of Ti_8_BTC MOG

Ti_8_BTC prepared according to the above procedure was initially subjected to a metalation process using four ruthenium compounds: RuCl_3_, [RuCl_3_(TPY)], [RuCl_2_(BPY)​(OH_2_)_2_]­Cl, and [RuCl_2_(PHEN)​(OH_2_)_2_]Cl (TPY: 2,2′:6′,2″-terpyridine; BPY: 2,2′-bipyridine; PHEN: 1,10-phenanthroline). The metalation was also performed with three more terpyridine metal complexes of cobalt­(II), copper­(II) and nickel­(II): [M­(TPY)­Cl_2_] (M: Co, Cu) and [Ni­(TPY)­Cl­(OH_2_)]­Cl. RuCl_3_ was employed as commercially obtained, while the rest of the complexes employed for the metalation were prepared following previously reported synthesis procedures and chemically characterized (see Sections S1 and S2 of the Supporting Information for more details).^25–27^ In the overall process, 4 g of Ti_8_BTC gel were immersed in a 30 mL ethanol solution with the corresponding metal source at a concentration of 2.2 mM, with the exception of the compound [RuCl_3_(TPY)] which was dissolved in 30 mL of DMF. The metal content was adjusted to 1 metal equivalent per octamer cluster. The metalation process was prolonged for 12 h at room temperature. The cleaning process to remove unreacted species was proceeded using successive ethanol exchanges in all cases except for [RuCl_3_(TPY)] that was cleaned as above-described for parent Ti_8_BTC. The obtained materials were coded as RuCl_3_@Ti_8_BTC, RuTPY@Ti_8_BTC, RuBPY@Ti_8_BTC, RuPHEN@Ti_8_BTC, CoTPY@Ti_8_BTC, NiTPY@Ti_8_BTC and CuTPY@Ti_8_BTC.

#### 2.1.5 Supercritical Drying Procedure

To process metal−organic gels as aerogels, an E3100 critical point dryer (Quorum Technologies), equipped with gas inlet, vent, and purge valves, as well as a thermal bath was used. The gels were immersed in liquid CO_2_ at 293 K and 50 bar for 1 h and the exchanged ethanol was removed through the purge valve. This procedure was repeated five times to ensure complete ethanol removal and substitution with liquid CO_2_. Then, the chamber temperature and pressure were raised to 313 K and 85–95 bar to achieve supercritical conditions of CO_2_. Once stabilized at 313 K, the chamber was gradually vented to atmospheric pressure. The resulting aerogels retained the shapes of the original parent gels. It is important to note that conventional solvent evaporation at room temperature (RT) or by heating in an oven causes significant microstructural contraction because of capillary forces generated during solvent evaporation, resulting in nonporous xerogels.

### 2.2 Characterization

#### 2.2.1 Chemical Characterization

Thermogravimetric analyses (TGAs) were carried out on a Mettler Toledo TGA/SDTA851 thermal analyzer, using a dynamic atmosphere of synthetic air (80% N_2_, 20% O_2_, flux of 50 cm^3^·min^–1^) and an alumina crucible. Thermal analysis curves were recorded from room temperature to 800 °C with a heating rate of 5 °C·min^–1^. Proton nuclear magnetic resonance (^1^H NMR) spectra were conducted using a Bruker AC-300 spectrometer, operating at 300 MHz, to determine the content of the organic ligand present in the samples analyzed. For this purpose, samples (50 mg) were digested in 2 mL of 1 M NaOH solution in deuterated water. The resulting solution was subjected to rapid digestion, maintained for 30 min. After this, fumaric acid (Sigma-Aldrich, +99%) was added as an internal standard (14 mg), and the solid residue was filtered off. The NMR spectrum was then recorded on the liquid fraction. Fourier transform infrared spectroscopy (FTIR) spectra of the samples were recorded with a resolution of 4 cm^–1^ in the region of 4000 to 400 cm^–1^ using a NICOLET-1550 FT-IR spectrometer. The FTIR spectra were recorded in reflectance mode directly on powder samples by coupling an attenuated total reflectance (ATR) module equipped with a diamond crystal. Powder X-ray diffraction (PXRD) measurements were taken at 20 °C in a PANalytical Xpert PRO diffractometer, equipped with a copper tube (λ = 1.5418 Å) and an automatic variable divergence slit. The range of 5–30° 2θ was covered with a step size of 0.02° and an acquisition time of 2.5 s per step. X-ray fluorescence (XRF) spectroscopy was used to determine the elemental metal content of the samples. A PANalytical wavelength dispersive X-ray fluorescence sequential spectrometer (WDXRF), model AXIOS, equipped with a Rh tube and three detectors (gas flow, scintillation and Xe-seal) was used for the analyses. Each sample was carefully ground in an agate mortar to ensure homogeneous particle distribution. Subsequently, the ground samples were placed in a sample holder for measurement. X-ray photoelectron spectroscopy (XPS) measurements were performed on a Phoibos 150 1D-DLD (SPECS) energy analyzer equipped with a Focus 500 monochromatic radiation source, an Al/Ag dual anode, and a SED-200 secondary electron detection system.

#### 2.2.2 Electron Microscopy

Scanning electron microscopy (SEM) analysis was carried out using a JEOL JSM-6400 electron microscope equipped with a secondary electron (SEI) detector and a backscattered electron (BSE) detector. Prior to SEM analysis, the samples were metallized with a 10 nm layer of gold. High-angle annular dark-field scanning transmission electron microscopy (HAADF−STEM) studies were done in a TECNAI G2 20 TWIN system operated at 200 kV and equipped with LaB_6_ filament, STEM unit with brightfield/darkfield detector and X-ray microanalysis unit (EDX). The samples were prepared by a dry dispersion onto a TEM copper grid (300 Mesh) covered by a holey carbon film. Additionally, transmission electron microscopy (TEM) measurements were performed using a FEI Titan Cubed G2 60–300 microscope equipped with a Schottky X-FEG field emission gun. The instrument includes HAADF (Fischione) and brightfield/darkfield detectors, a Super-X EDX detector (ChemiSTEM technology with ESPRIT software), and a Gatan Quantum ER/965 energy filter.

#### 2.2.3 N_2_ Adsorption Measurements

Physical nitrogen adsorption isotherms were recorded at 77 K using a Quantachrome Autosorb iQ MP analyzer. Prior to data collection, the samples were degassed at 150 °C under high vacuum (ca. 10^–5^ bar) for 6 h. The determination of the specific surface area was performed using the Brunauer–Emmett–Teller (BET) model.^28^ To avoid ambiguity when reporting the BET surface area of materials potentially containing micropores we defined the pressure range for the data fitting according to the three consistency criteria proposed by Rouquerol et al.: (1) the pressure range selected should have values of *V*(1 – *p*/*p* ^o^) increasing with *p*/*p* ^o^, (2) the points used to calculate the BET surface area must be linear with an upward slope in such a way that the linear regression must yield a positive *y*-intercept (i.e., positive *C* value) and (3) the *p*/*p* ^o^ value corresponding to *V*_m_ should be within the BET fitting range.^29^ The micropore volume of the samples was estimated according to the *t*-plot method,^30^ and the pore size distribution (PSD) was determined using the Barrett–Joyner–Halenda (BJH) method.^31^

#### 2.2.4 Optical Characterization

Diffuse reflectance UV–Vis spectroscopy (DRS-UV–Vis) analysis was performed using a UV–visible–NIR JASCO 770 V spectrometer, equipped with an integrating sphere coated with Spectralon and offering a spectral resolution of 1 nm. The spectra were recorded in reflectance mode in the 300–800 nm range at a 600 nm·min^–1^ scan rate. Spectralon is used as standard for 100% reflectance measurements due to its high diffuse reflectance over the ultraviolet, visible, and near-infrared regions, while internal attenuators are employed to establish zero reflectance and minimize background noise. Photoluminescence (PL) spectroscopy measurements in fluorescence mode were performed on a Varian Cary Eclipse (Agilent Technologies) optical spectrometer, equipped with a Xenon flash lamp of 450 W, a monochromator and a 90°—located photomultiplier (PMT). The measurements performed in emission scan mode were recorded in the 370–700 nm range using an excitation wavelength of 365 nm, 600 V PMT, and a 600 nm·min^–1^ scan rate.

#### 2.2.5 X-ray Absorption Fine Structure Spectroscopy

Near-edge X-ray absorption fine structure (NEXAFS) measurements at the Ti L_2,3_-edges were carried out at the B07-B beamline (VerSoX) of Diamond Light Source (UK).^32^ Powder samples were prepared by gently pressing them into indium foil, ensuring good electrical conductivity and minimizing the risk of contamination and were mounted in the ES-2 chamber, which is separated from the beamline by a silicon nitride membrane. The incident X-ray beam, delivered from a bending magnet and monochromatized by a plane grating monochromator (PGM), was focused to a spot size of approximately 80 μm × 200 μm (fwhm) at the sample position. The specimen chamber pressure was maintained at 10^–7^ mbar during measurements. All measurements were performed in total electron yield (TEY) mode, which is well-suited for surface-sensitive detection. The spectra were normalized to the beamline transmission by dividing by the *I*_0_ measurement and then normalized to the postedge region.

### 2.3 Photocatalytic Hydrogen Evolution Experiments

The ability of the developed materials to act as photocatalysts of the hydrogen evolution reaction (HER) was analyzed using a reactor equipped with a monochromatic UV lamp (Hepatochem, λ_exc_ = 365 nm, nominal power = 10 W), delivering an effective power of 112 mW and an intensity of 142.7 mW·cm^–2^ (measured at 8 cm). For the semicontinuous monitoring of hydrogen production the system also included mass flow controllers (MFC, Bronkhorst) and a gas chromatograph (GC-2030 Shimadzu) equipped with a barrier discharge ionization detector (BID) and a SH-Msieve 5A column (length: 30 m, internal diameter: 0.32 mm and internal film thickness: 30 μm) as shown schematically in Figure 1.

**Figure 1:**
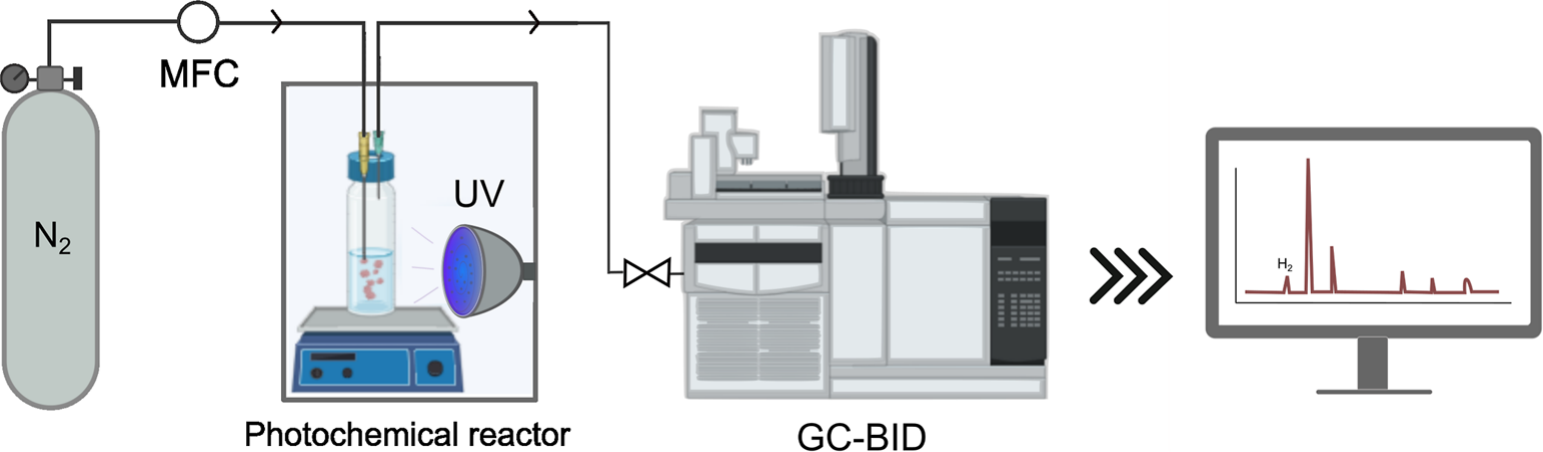
Scheme of the setup used for the photocatalytic HER experiments. The photochemical reactor was an opaque chamber isolated from external light.

In the standard procedure, 5 mg of each of the prepared MOAs were placed in a 15 mL borosilicate glass vial (Chromacol 10-SV, Fisher) together with 8 mL of a 0.1 M aqueous solution of triethanolamine (TEOA) (pH adjusted to 7 with 2 M HCl) acting as a sacrificial agent (electron donor). Experiments were also performed with greater catalyst mass (10 and 20 mg) to analyze the effect of the catalyst concentration. Each reaction vial was sealed, sonicated for 10 min and connected to the gas line to purge it for 20 min with N_2_ gas (20 cm^3^·min^–1^) before irradiation. After that, the reactor vial was set at a distance of 8 cm from the light-source (previously optimized, Figure S22 of Supporting Information) and irradiated under continuous stirring. The headspace of the vial was constantly purged with N_2_ at a flow rate of 5 mL·min^–1^, controlled by the mass flow controller. The outflow was fed to the GC-BID, and samples were automatically injected every 15 min for analysis. Hydrogen evolution rates (μmol·h^–1^) were calculated from the measured H_2_ concentration in the purged gas and the purge gas flow rate. The analytical calibration was performed using certified standard gas H_2_ (1000 ± 20 mol-ppm in nitrogen from AirLiquide) which was further diluted with nitrogen for the preparation of the calibration standards. The obtained limit of quantification (LoQ) and coefficient of determination (*R* ^2^) were 2.0 ppm and 0.9999, respectively. Each experiment was performed twice and the results are presented as the mean value together with the standard deviation.

## 3 Results and Discussion

### 3.1 Chemical Characterization of Ti_8_BTC

In this section, the main chemical characteristics of synthesized Ti_8_BTC MOA are described. It should be noted that the estimation of its chemical formula was carried out following previously established procedures^33^ and considering data extracted from chemical and thermogravimetric analysis, as detailed below.

The formation of Ti_8_BTC occurs via ligand exchange reaction of Ti_8_BNZ with the polycarboxylic ligand H_3_BTC (Figure 2). The polymerization processes and the formation of higher-dimensional metal−organic polymers are generally thermodynamically favored.^34^ Specifically, the substitution of terminal BNZ ligands by bridging BTC ligands enhances intercluster interactions, transforming relatively weak noncovalent forces such as hydrogen bonding and dispersion into stronger coordination bonds. At the same time, each bridging BTC ligand displaces multiple terminal ligands, leading to a net increase in entropy of the system, which further stabilizes the extended gel network. This specific reaction requires the use of DMF to dissolve the metal complex. It should be noted that DMF is a common solvent in the synthesis of MOFs,^35^ which tends to partially decompose into formate (FOR) and dimethylammonium (DMA), and consequently the presence of formato as a ligand is common in many MOFs of group 4 metals (titanium, zirconium, and hafnium) together with the main organic ligand.^36^ In fact, ^1^H NMR measurements of the MOA suggest that the ligand exchange reaction involves a partial substitution of the benzoato ligands by the polycarboxylic bridging ligand and by formate anions (a second blocking ligand) (See Section S3.1 of Supporting Information).

**Figure 2:**
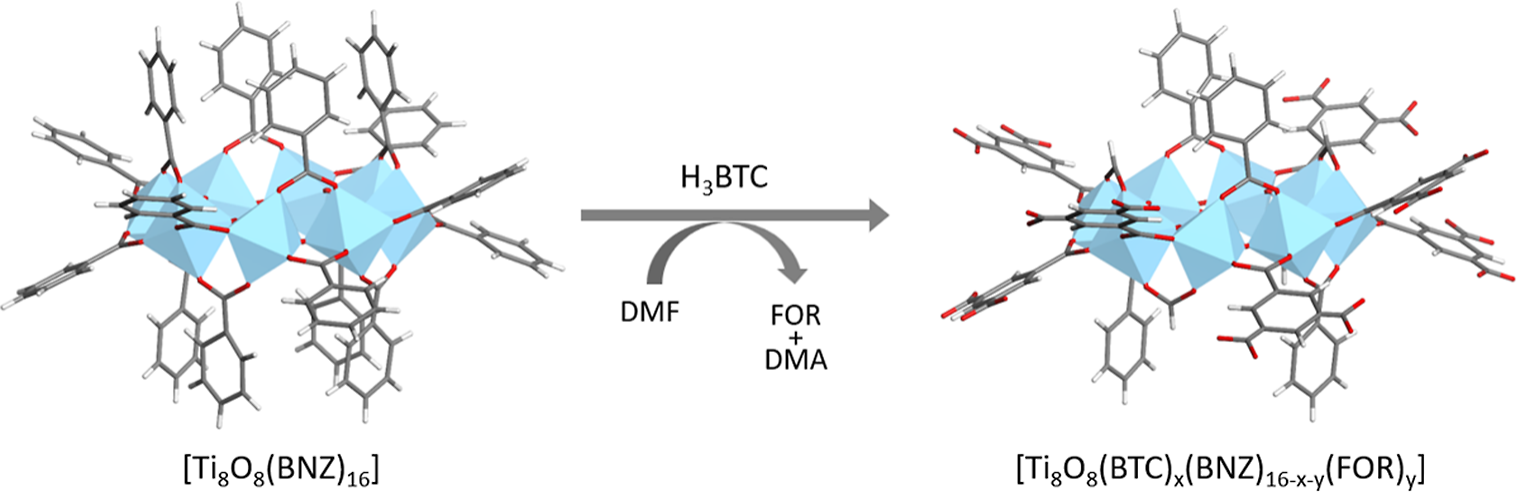
Reaction scheme for the ligand exchange on Ti_8_BNZ to afford Ti_8_BTC depicting the partial substitution of benzoato ligands by benzene-1,3,5-dicarboxylato and formato at the cluster.

According to the thermogravimetric analysis data (Section S3.2 of Supporting Information), the molar mass of the materials (*M*_MOA_) after moisture release is 1915.6 g·mol^–1^, normalized to the Ti_8_O_8_ cluster. Note that the molar mass of the material can be expressed based on the molar mass (*M*) and the stoichiometry of each component (*n*_*i*_: coefficient of ligand *i*; *n*_solv_: coefficient of the trapped solvent), as represented in eq 1. The stoichiometric coefficient of each component has been determined from the species identified and quantified in the chemical analysis, which results in the formula {[Ti_8_O_8_​(BTC)_4.80_​(BNZ)_1.44_​(FOR)_2.84_]·​(EtOH)_0.57_·(BuOH)_1.31_}_*n*_. At this point, eq 1 can be converted into eq 2 to calculate a molecular mass value of 1933.4 g·mol^–1^, which implies a minor deviation with respect the experimental value (|σ|: 0.92%) and with the expected decomposition products of the thermogravimetric evolution (see Table S2 of Supporting Information). It should be noted that the calculated molecular weight considers carboxylic protons as estimated below for BTC, however, omitting them does not result in significant changes in the overall molar mass. In addition, the molar mass of the product formed at 350 °C (1735.3 g·mol^–1^) matches rather well the formula expected after the release of solvent molecules and formato ligands. In this sense, at temperatures of 200–300 °C formate anions are released to yield hydroxide groups^35^ ([Ti_8_O_8_(OH)_2.84_(BTC)_4.80_(BNZ)_1.44_], 1730.5 g·mol^–1^) which at temperatures close to 350 °C tend to condense into oxide ions (releasing water) to afford the expected mean formula [Ti_8_O_9.42_​(BTC)_4.80_​(BNZ)_1.44_] (1704.9 g·mol^–1^, |σ|: 1.78%).

1 $$M_{{Ti}_{8}BTC}=M_{cluster}+\sum n_{i}\cdot M_{i}+\sum n_{solv}\cdot M_{solv}$$

$$M_{{Ti}_{8}BTC}={M_{cluster}+n}_{BTC}\cdot M_{BTC}+n_{BNZ}\cdot M_{BNZ}+n_{FOR}\cdot M_{FOR}+n_{MeOH}\cdot M_{MeOH}+n_{BuOH}\cdot M_{BuOH}$$ 2

It is important to note that the coordination of the polycarboxylic ligand (H_3_BTC) can take place via one, two, or even three carboxylate groups. This implies that a variable number of carboxylic groups can remain free (or uncoordinated) as represented in the formula as C_6_H_3_(COO)_3–*x*_(COOH)_*x*_. The number of free carboxylic groups (*x*) plays a crucial role in the charge of the complex. Since the charge of the ligands (*q*_*i*_: charge of ligand (*i*)) around the cluster must counterbalance the charge of the cluster (eq 3), in the present case the number of free carboxylic groups per polycarboxylic ligand can be derived using eq 4 and defining the average charge of this ligand as *q*_BTC_ = −(3 – *x*). The charges of benzoate and formate are set by their single carboxylic group (*q*_BNZ_ = *q*_FOR_ = −1) while the charge of the oxo-cluster (*q*_cluster_) is equal to +16. As a result, the estimated number of free carboxylic groups per BTC ligand is 0.56. Therefore, the average molecular formula of the coordination structure can be written as [Ti_8_O_8_​{C_6_H_3_(COO)_2.44_​(COOH)_0.56_}_4.80_​(C_6_H_5_COO)_1.44_​(HCOO)_2.84_]_*n*_.

3 $$q_{cluster}=-\sum_{i=1}^{m}n_{i}\cdot q_{i}$$

$$q_{cluster}=-\left(n_{BTC}\cdot q_{BTC}+n_{BNZ}\cdot q_{BNZ}+n_{FOR}\cdot q_{FOR}\right)$$ 4

Furthermore, according to eq 5, the analysis of the coordination positions available in the Ti_8_O_8_ cluster (*D*_cluster_ = 32) and the donor atoms of the ligands (*D*_*i*_: donor atoms of ligand *i*) further supports the proposed formula. In this regard, in the case of the precursor (Ti_8_BNZ: [Ti_8_O_8_(BNZ)_16_]) used in the ligand exchange reaction, it is clear that the 16 BNZ ligands with μ-carboxylato-κ*O*:κ*O*′ coordination mode (*D*_BNZ_ = 2) satisfy the available coordination positions of the cluster. Similarly, the balance of coordination positions in Ti_8_BTC can be calculated using eq 6 and assuming the same coordination mode for the carboxylate groups present, which implies that each capping ligand provides two donor atoms (*D*_BNZ_ = *D*_FOR_ = 2) while the proportional number of donor atoms per polycarboxylic ligand corresponds to *D*_BTC_ = 2­(3 – *x*) = 4.88. Therefore, the calculated total number of potential donor atoms for the ligand ensemble is 31.96, aligning reasonably well with the available coordination sites of the cluster, as occurs for the Ti_8_BNZ precursor.

5 $$D_{cluster}=\sum n_{i}\cdot D_{i}$$

$$D_{cluster}=n_{BTC}\cdot D_{BTC}+n_{BNZ}\cdot D_{BNZ}+n_{FOR}\cdot D_{FOR}$$ 6

It should be added that in MOFs, the bridging ligand vacancies can be replaced by blocking ligands and/or hydroxyl groups (or hydroxyl/water pairs),^33^ but according to the analysis described above the presence of coordinated hydroxyl/water pairs appears to be negligible after the ligand exchange reaction.

The characterization of the MOA was further supported by the FTIR analysis data (Section S3.3 of Supporting Information). The assignment of the main vibrational modes is presented in Table S3.^37,38^ For comparative purposes and to focus on the main vibration bands ascribed to carboxylate groups, the FTIR spectrum of Ti_8_BTC within the 2000–1000 cm^–1^ wavenumber range is compared with the spectra of the precursor (Ti_8_BNZ) and the acidic form of the bridging ligand (H_3_BTC) (Figure 3). The free carboxylic groups (−COOH) that remain uncoordinated at Ti_8_BTC are distinguishable by the band at 1705 cm^–1^ related to the stretching vibration of the carbonyl group. This band is very intense in H_3_BTC, while is understandably absent in the Ti_8_BNZ precursor, as it lacks protonated carboxylic groups. This feature allowed the estimation of the total and accessible −COOH groups in Ti_8_BTC through a cumulative standard addition experiment combined with FTIR monitoring the ν­(C=O) band at 1705 cm^–1^. In this method, incremental amounts of H_3_BTC were used as internal standard to minimize matrix effects and ensure consistent response of the carbonyl vibration band. The intercept of the standard addition plot was used to determine the total carboxylic acid content, yielding a value of ca. 2 mol of COOH per mol of MOA, which implies a reasonably good agreement with the estimation previously obtained from TGA, ^1^H NMR, and charge balance analyses. Additionally, the MOA was treated with ammonia to neutralize the surface-accessible carboxylic groups forming (NH_4_^+^)­(COO^–^) ensembles, and the remaining unreacted −COOH groups were quantified following an analogous procedure. The difference between both determinations corresponds to the fraction of surface-accessible carboxylic acids, yielding 0.6 mol of COOH per mol of MOA, i.e., approximately the 30% of the total −COOH sites (see Section 3.3 in the Supporting Information for full details).

**Figure 3:**
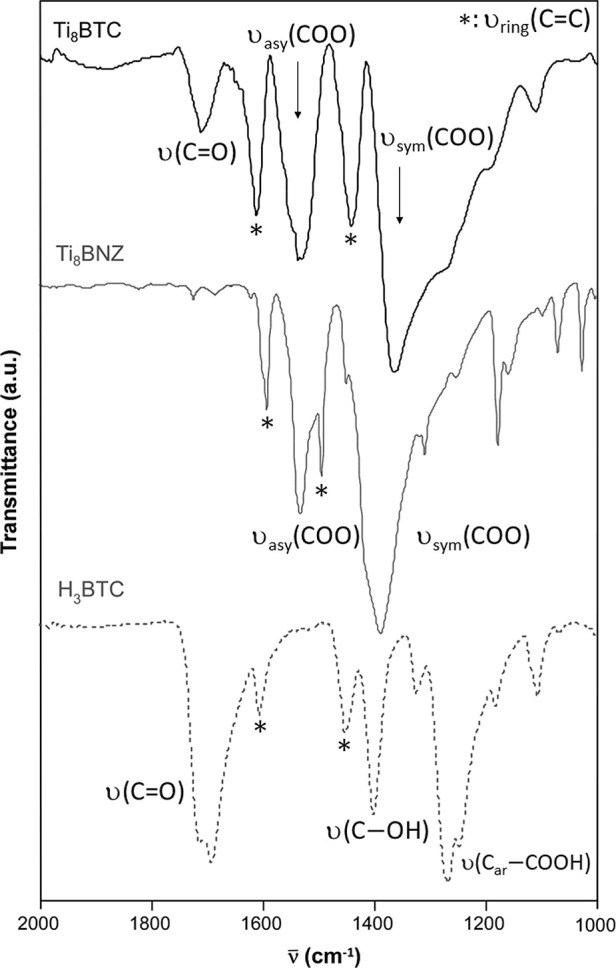
FTIR spectra of Ti_8_BTC MOA compared to the precursor Ti_8_BNZ and the acidic form of the H_3_BTC bridging ligand.

On the other hand, the characteristic bands of the coordinated carboxylate groups, corresponding to the symmetric and antisymmetric stretching vibrations, appear in Ti_8_BTC at 1540 and 1375 cm^–1^, respectively. These bands can also be found in Ti_8_BNZ at similar wavenumbers (1530 and 1380 cm^–1^). Lastly, both the Ti_8_BNZ precursor and the product of the metathesis reaction (Ti_8_BTC) show bands around 580 and 450 cm^–1^, which are related to the vibration of Ti–O bonds in the octanuclear cluster.^39^

The chemical characterization was completed by PXRD analysis of Ti_8_BTC MOA (See Figure S9). Although the material lacks crystallinity, it shows a broad peak at 2θ = 5–8°, which corresponds to a spacing of 11–18 Å and can be ascribed to the average radial distribution of neighboring titanium clusters within the coordination polymer.

### 3.2 X-ray Absorption Fine Structure Spectroscopy

Soft X-ray absorption spectroscopy (NEXAFS) was carried out at the Ti L_2,3_-edge to further support the prevalence of the octanuclear titanium-oxo cluster in Ti_8_BTC after the ligand exchange process performed using Ti_8_BNZ as precursor.

As shown in Figure 4, the normalized absorption spectra of Ti_8_BNZ and Ti_8_BTC display a striking similarity in both energy position and spectral shape. According to the theoretical framework described by de Groot et al.,^40^ the spectral profile at the L-edge is mainly dominated by final-state effects, which are highly sensitive to the local symmetry, oxidation state, and ligand–field interactions around the absorbing atom. In this context, soft X-ray absorption spectroscopy probes the unoccupied electronic states by promoting core electrons (in this case, 2p electrons of titanium) to unoccupied 3d orbitals, resulting in characteristic spectroscopic features that reflect the local electronic environment and coordination geometry of the metal center. Specifically, the main peaks observed correspond to the L_3_ (2p_3/2_ → 3d) and L_2_ (2p_1/2_ → 3d) absorption edges, which arise due to spin–orbit splitting of the Ti 2p core level. Each edge further exhibits a characteristic splitting into t_2g_ and e_g_ components, reflecting the octahedral ligand field around the Ti­(IV) centers.^41^ The close match between the two spectra, with no evidence of new features or significant shifts in energy, strongly supports the retention of the original Ti_8_ cluster motif in the final Ti_8_BTC framework. The preservation of the characteristic four-peak structure indicates that the local electronic environment of the titanium centers remains largely unchanged, suggesting minimal perturbation during metal−organic gel formation.

**Figure 4:**
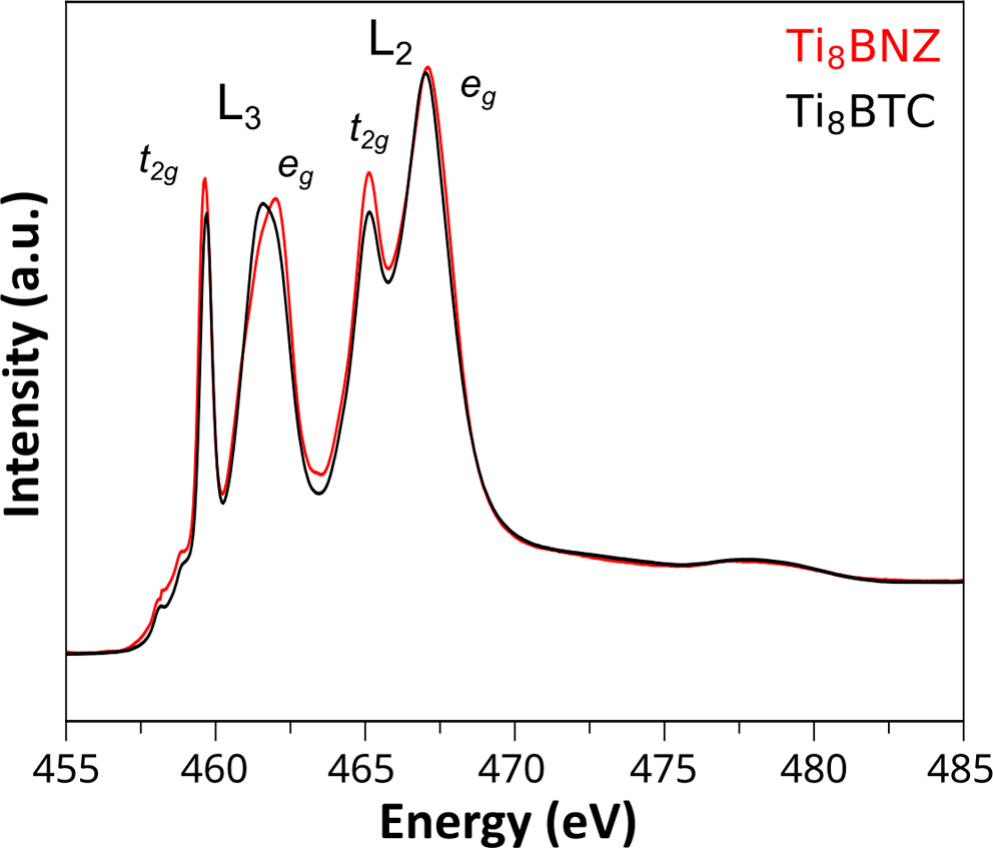
Normalized Ti L_2,3_-edge experimental NEXAFS spectra of Ti_8_BNZ and Ti_8_BTC with assignment of the main features associated with Ti­(IV) octahedral centers.

While the overall spectral profiles are remarkably similar, small differences in intensity and peak position are observed. Specifically, a slight change in the relative intensity of the L_3_-t_2g_ and L_2_-t_2g_ features, along with a minor energy shift of the L_3_-e_g_ peak can be discerned. These subtle variations are consistent with minor distortions in the local octahedral geometry and with small modifications in the extended coordination environment of the Ti centers upon ligand exchange. In Ti_8_BNZ, the benzoato ligands act as capping units, while in Ti_8_BTC, the tritopic BTC ligands interconnect the clusters into the gel matrix. This change in connectivity likely introduces small deviations in the Ti–O bond angles or distances, affecting the ligand field strength and thereby the crystal field splitting.^40–42^ In addition, according to Krüger,^42^ spectral features in the L_3_-e_g_ region are particularly sensitive to structural effects beyond the first coordination shell, reflecting the structure on a length scale of approximately 1 nm. Their work demonstrates that fine structure variations can arise from changes in the extended framework arrangement rather than from direct modifications to the TiO_6_ octahedra themselves, or at least have a higher contribution. Therefore, the spectral variations observed in the L_3_-e_g_ peak between Ti_8_BNZ and Ti_8_BTC can be attributed to the different extended connectivity patterns (discrete vs polymer) rather than significant changes to the individual cluster structure.

### 3.3 Microstructural Characterization of Ti_8_BTC

The porous characteristics of the Ti_8_BTC aerogel were initially evaluated through nitrogen adsorption measurements at 77 K. The isotherm obtained (Figure 5a) exhibits a Type II/IV curve according to the IUPAC classification,^43^ with a narrow hysteresis loop at high relative pressures (*p*/*p* ^o^ ≈ 0.8–0.9), as a result of the combination of macropores and mesopores. The plot of the BJH cumulative pore volume (Figure 5b) shows a polydisperse pore sizes distribution as expected for a microstructure comprised by the stochastic cross-linking of the metal−organic particles. The quantitative analysis extracted from the adsorption data is shown in Table 1. Fitting of the adsorption data to the BET equation led to a surface area value of 612 m^2^·g^–1^, while the analysis of the total pore volume yielded a value of 2.86 cm^3^·g^–1^ (pores ≤ 180 nm). In this regard, according to the data derived from the *t*-plot analysis, the microporosity contributes only a minor portion of the total surface area and pore volume of the materials. Although the BET surface area is moderate compared to crystalline Ti-MOFs, the presence of large meso- and macropores results in hierarchical porosity that facilitates the diffusion of reactants and products, which is often beneficial for catalytic performance, as has been demonstrated in previous studies.^17^

**Figure 5:**
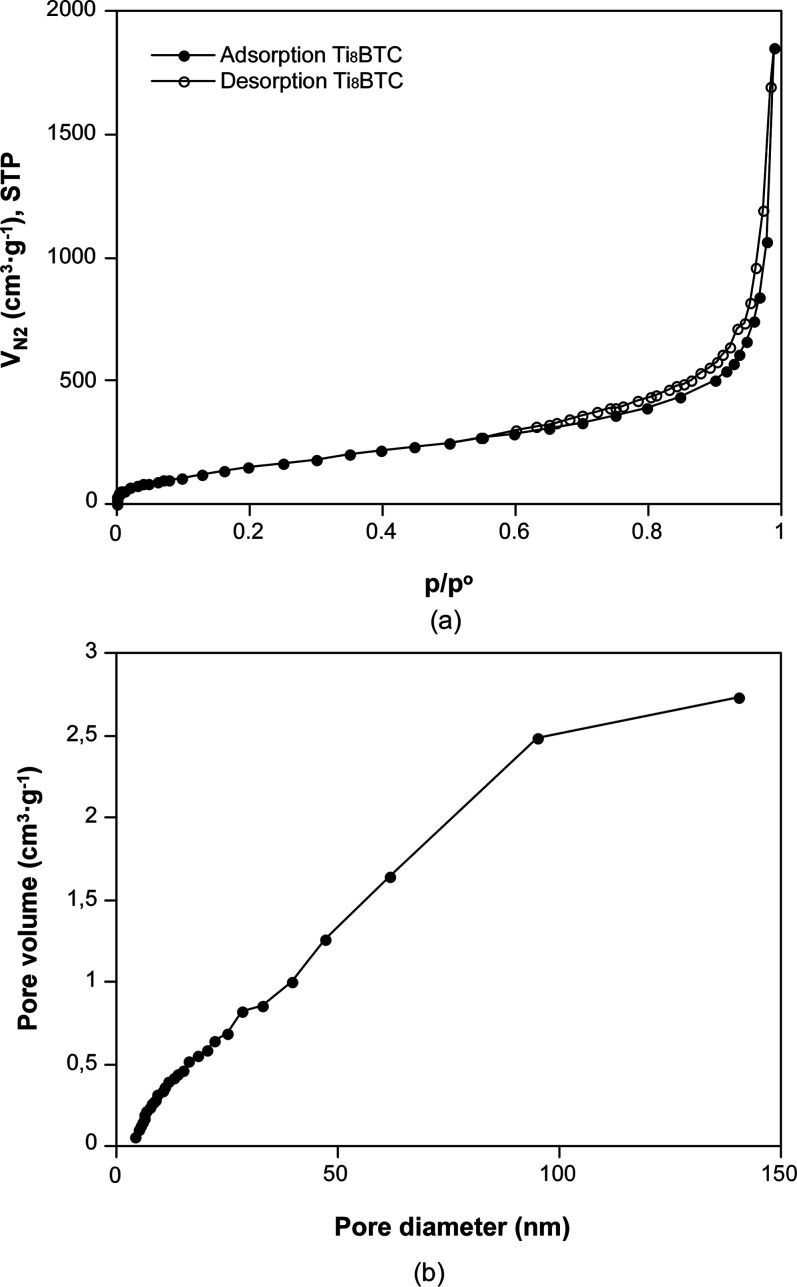
(a) Nitrogen adsorption isotherm (77 K) and (b) BJH cumulative pore size distribution for Ti_8_BTC MOA.

**Table 1:** Surface Area and Pore Volume Extracted Data from the N_2_ (77 K) Adsorption Isotherm for Ti_8_BTC MOA^a^

*S*_BET_ (m^2^·g^–1^)	*S*_micro_ (m^2^·g^–1^)	*S*_ext_ (m^2^·g^–1^)	*V*_T_ (cm^3^·g^–1^)	*V*_micro_ (cm^3^·g^–1^)	*V*_ext_ (cm^3^·g^–1^)
612	49	563	2.86	0.03	2.83

a *S*_BET_ corresponds to BET specific surface area. *S*_micro_ (micropore surface area) and *V*_micro_ (micropore volume) are determined from the *t*-plot calculation. *S*_ext_ (external surface area) is obtained by subtracting the microporous contribution to the total surface area. *V*_T_ (total pore volume) is calculated for pores ≤180 nm.

The microstructural characteristics of Ti_8_BTC were further investigated using scanning electron microscopy (SEM) and high-angle annular dark-field scanning transmission electron microscopy (HAADF−STEM). Secondary electron (SE) SEM and HAADF−STEM images (Figure 6a,b) reveal that the aerogel sample consists of nanoscopic particles (<50 nm) that partially sinter to form larger aggregates. These aggregates create a solid network characterized by high porosity and a broad polydispersity in pore size (approximately 10–200 nm), consistent with the previously described N_2_ adsorption data. These microstructural features are typical for such materials, resulting from the stochastic cross-linking of metal−organic nanoparticles during gel formation.^15,44^ At higher magnification, high-resolution HAADF−STEM images (Figure 6c,d) show a framework composed of annular motifs in which brighter areas correspond to more electrodense parts of the coordination polymer. The diameter distribution analysis of that annular motifs (Figure 6e) provides a mean value of 1.32 nm, which fit well with the size of the octanuclear secondary building unit comprised by the octanuclear Ti­(IV) cluster and the linked donor O atoms (Figure 6f).

**Figure 6:**
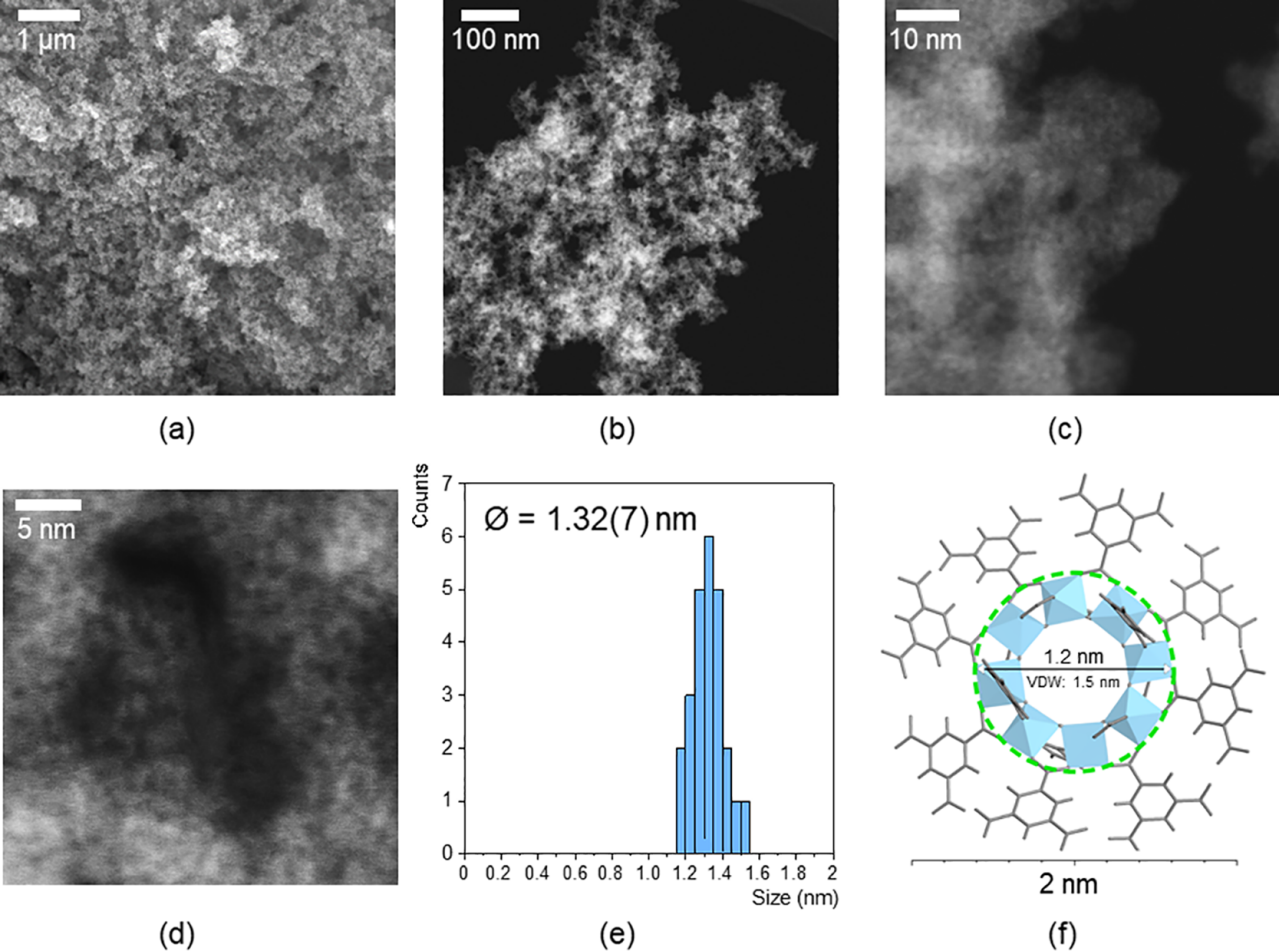
(a) Secondary electron (SE) SEM micrographs, (b–d) HAADF−STEM images, (e) diameter distribution analysis of annular motifs found in (d), and (f) secondary building unit comprised by the octanuclear Ti­(IV)-oxo cluster and the linked donor O atoms.

### 3.4 Post-synthetic Modifications

The metalation process of Ti_8_BTC entails a post-synthetic step involving the introduction of an equivalent amount of dopant metal (M_D_: Ru, Ni, Co, or Cu) per Ti-oxo octanuclear cluster into the reaction environment. Initially, ruthenium­(III) sources, specifically RuCl_3_ and RuCl_3_(L_N_) complexes, where L_N_ represents 2,2’:6′,2″-terpyridine (TPY), 2,2′-bipryridine (BPY) and 1,10-phenanthroline (PHEN), were employed for the metalation reaction to assess the influence of different N-ligands. Thereafter, three more Ti_8_BTC samples underwent metalation using M_D_(TPY) complexes (with M_D_ being Ni­(II), Co­(II), or Cu­(II)).

The successful anchoring of the metal complexes was visually evident through color changes in comparison with the initially white Ti_8_BTC material. Specifically, RuCl_3_@Ti_8_BTC exhibited a light pink hue; RuTPY@Ti_8_BTC, a dark purple color; RuBPY@Ti_8_BTC, a dark blue tone; RuPHEN@Ti_8_BTC, a yellow color; CoTPY@Ti_8_BTC, a light brown hue; NiTPY@Ti_8_BTC, a turquoise color; and CuTPY@Ti_8_BTC shows light blue turquoise (as illustrated in Figure 7).

**Figure 7:**
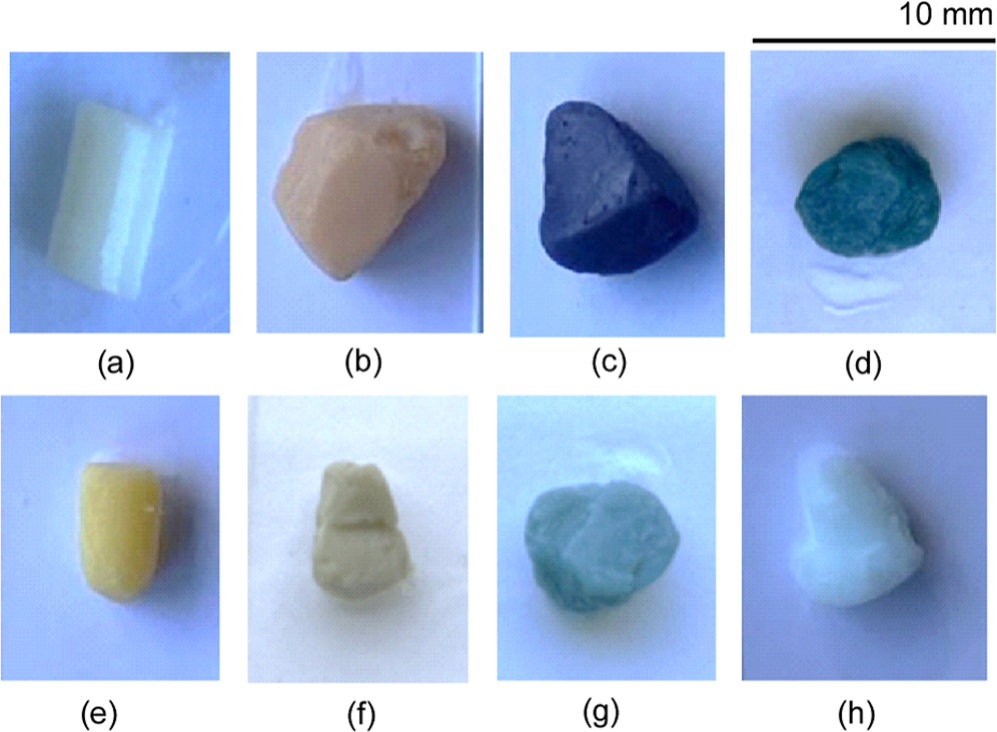
Pictures of Ti_8_BTC monoliths before and after doping: (a) Ti_8_BTC, (b) RuCl_3_@Ti_8_BTC, (c) RuTPY@Ti_8_BTC, (d) RuBPY@Ti_8_BTC, (e) RuPHEN@Ti_8_BTC, (f) CoTPY@Ti_8_BTC, (g) NiTPY@Ti_8_BTC, and (h) CuTPY@Ti_8_BTC.

The XRF analysis data gathered in Table 2 confirm the presence of the dopants in amounts lower than those established in the reaction (1:1 for M_D_/Ti_8_O_8_). It should be noted that, the number of accessible free carboxylic groups (−COOH: 0.6 per cluster) is sufficient to incorporate all the dopant metal from the reaction mixture. Nevertheless, according to the values observed in Table 2, the post-synthetic reaction had a low/medium yield, despite the affinity of the selected metals for coordinating with this functional group.^45^ This behavior can be partly attributed to the limited diffusion of the dopant complex into the metal−organic particle, restricting anchoring to the carboxylic groups available on the external surface. It is also worth noting that the amount of incorporated ruthenium is significantly lower compared to cobalt, copper, and nickel complexes. This may be ascribed to the higher inertness of ruthenium (a second–row transition metal) which makes the ligand exchange process required for the anchoring more challenging than in the case of cobalt, nickel and copper.

**Table 2:** Relative Mass Content and Atomic Ratios for Titanium and Dopant Metals (M_D_: Ru, Co, Ni, and Cu) Determined by XRF Spectroscopy

samples	Ti (% wt.)	M_D_ (% wt.)	Ti_8_/M_D_ (at.)
RuCl_3_@Ti_8_BTC	99.20	0.80	1:0.031
RuTPY@Ti_8_BTC	99.59	0.41	1:0.016
RuBPY@Ti_8_BTC	99.63	0.37	1:0.014
RuPHEN@Ti_8_BTC	99.62	0.38	1:0.015
CoTPY@Ti_8_BTC	97.99	2.01	1:0.13
NiTPY@Ti_8_BTC	97.81	2.19	1:0.15
CuTPY@Ti_8_BTC	97.99	2.01	1:0.12

Doped aerogels were further analyzed by X-ray photoelectron spectroscopy (XPS) measurements. The high-resolution spectrum of each of the elements observed with their characteristic components are presented in Figure 8 for RuTPY@Ti_8_BTC, as a representative case of the post-synthetically modified samples (XPS spectra of the rest of the doped systems are presented in Section S3.8 of the Supporting Information). This analysis allowed to identify the distinctive 2p peaks of titanium­(IV) and their three satellite peaks, as observed for Ti_8_O_8_ based MOFs.^46^ Furthermore, the peaks at 284.6 and 280.5 eV are attributable to Ru 3d_3/2_ and 3d_5/2_ levels of Ru­(III), respectively, while bands at 401.4 and 398.7 eV correspond to N 1*s* signals from the terpyridine ligand. The intensity of these bands is small due to the low loading of the ruthenium complex in the sample. Finally, expected C 1s and O 1s bands coming from the organic linkers are observed. In the case of carbon, two C 1s bands at 288.4 and 284.6 eV are ascribed to O–C=O and C–C/C–H carbon-type, respectively. Finally, the band at 531.6 eV for O 1*s* comes from the contribution of carboxylic, oxide and hydroxide ligands.^47^

**Figure 8:**
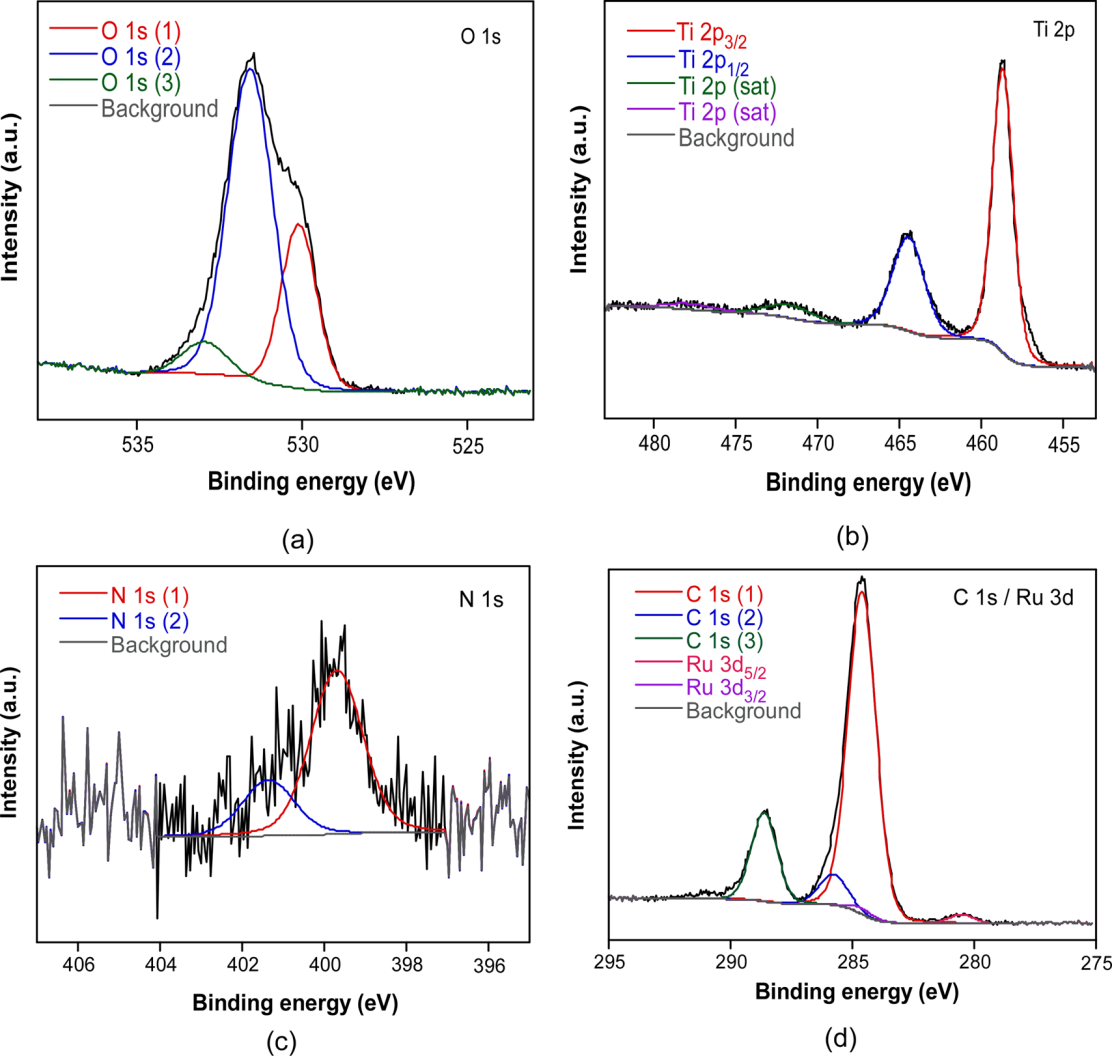
High-resolution XPS spectrum for (a) O 1s, (b) Ti 2p, (c) N 1s, (d) C 1s and Ru 3d for RuTPY@Ti_8_BTC.

HAADF−TEM images and element mapping analysis show a fine and homogeneous distribution of metal dopants throughout the metal−organic aerogel, which is consistent with a situation in which the metal complex is spread over the metal−organic polymer instead of clustering within the microstructural pores. Figure 9 shows the images corresponding to RuTPY@Ti_8_BTC and CuTPY@Ti_8_BTC, while additional mappings for other samples are provided in Section S3.5 of Supporting Information (Figures S10–S13).

**Figure 9:**
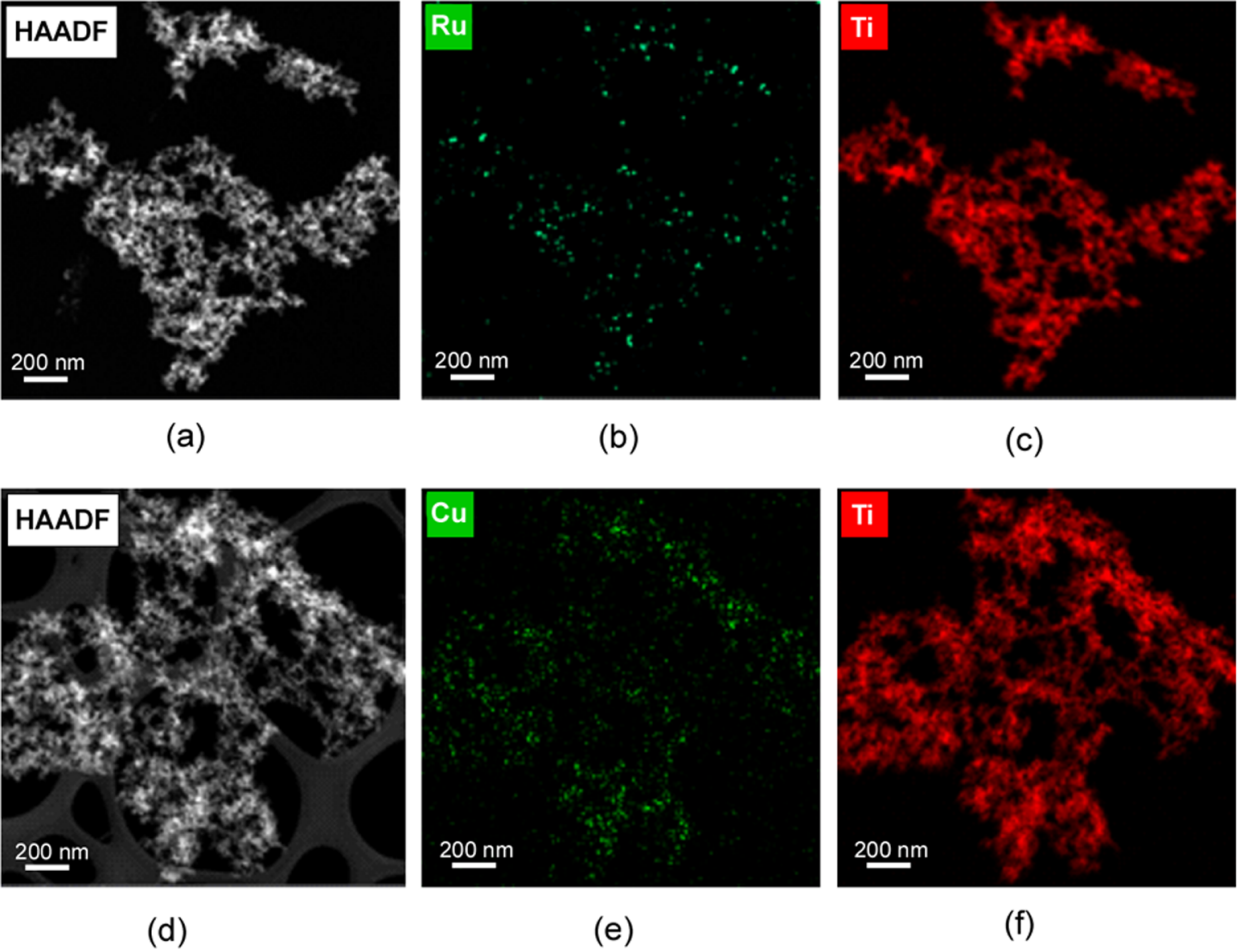
(a) HAADF−TEM micrograph and elemental mapping for (b) Ru and (c) Ti taken on RuTPY@Ti_8_BTC sample and (d) HAADF−TEM micrograph and elemental mapping for (e) Cu and (f) Ti taken on CuTPY@Ti_8_BTC.

SEM images of the post-synthetically modified sample supports also that the aerogels retain the overall microstructure of the parent Ti_8_BTC (Figure S14). Accordingly, the specific surface areas determined by the BET method for the ruthenium samples (530–588 m^2^·g^–1^) are similar to that of the undoped Ti_8_BTC material, suggesting that ruthenium complex incorporation does not significantly alter the porous structure. However, in the cases of CoTPY@Ti_8_BTC, CuTPY@Ti_8_BTC, and NiTPY@Ti_8_BTC, a noticeable decrease in surface area is observed upon doping (373–476 m^2^·g^–1^). This reduction could be attributed to partial pore blockage or agglomeration caused by the introduction of the metal complexes (see the isotherms in Figure 10). Nitrogen adsorption isotherms of the rest of the doped systems can be seen in Section S3.7 of the Supporting Information.

**Figure 10:**
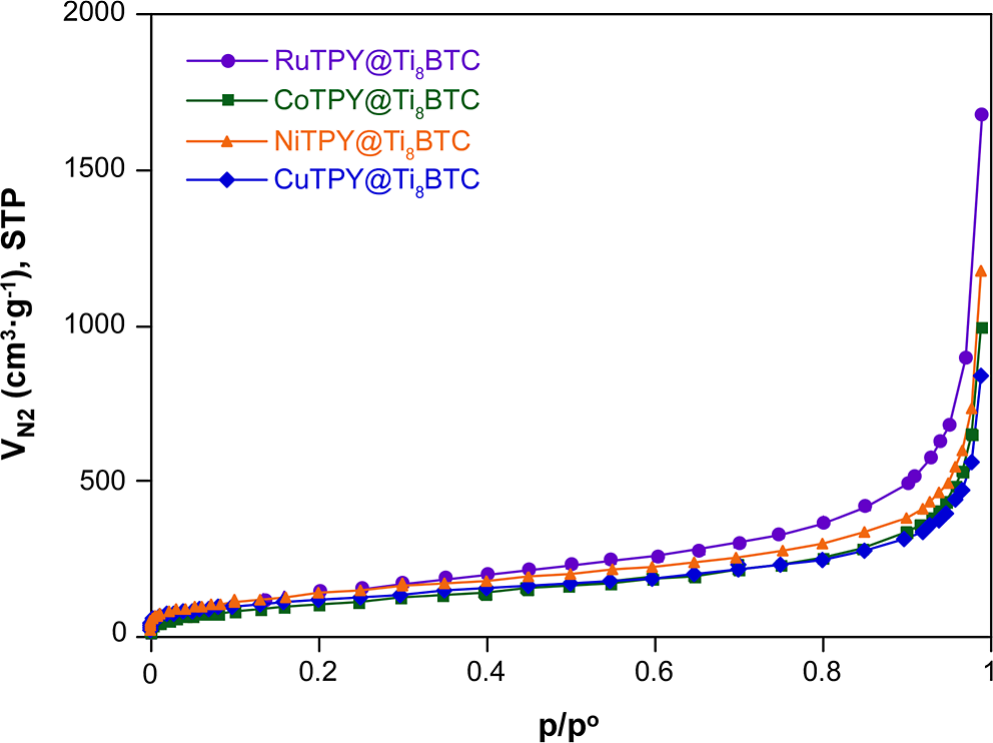
Nitrogen adsorption isotherm (77 K) for RuTPY@Ti_8_BTC, CoTPY@Ti_8_BTC, NiTPY@Ti_8_BTC, and CuTPY@Ti_8_BTC.

### 3.5 Optical Properties

In order to analyze the optical properties of the materials prior to the photocatalysis experiments, they were studied by UV–vis diffuse reflectance spectroscopy (DRS). The spectra were transformed using the Kubelka–Munk function, *F*(*R*) = (1 – *R*_∞_)^2^/(2·*R*_∞_) (*R*_∞_ = *R*_sample_/*R*_reflectance_),^48,49^ to approximate the variation of absorption with wavelength. The transformed spectra for Ti_8_BTC and Ru- and Cu-terpyridine functionalized samples are presented in Figure 11 (the rest of the spectra are provided in Section S3.9 of the Supporting Information). As can be seen, the Ti_8_BTC presents an intense band in the ultraviolet region (λ_max_ = 330 nm) whose onset of absorption is close to 400 nm (3.10 eV). Values for the energy of the band gap (*E*_g_) were estimated by fitting the spectrum to the Tauc equation, (*ah*v)^1/*n*^ = *A*(*hn* – *E*_g_), where α represents the absorption coefficient, *A* is a constant term, *h*ν is the photon energy and *n* is a parameter related to the type of band transition (*n* = 0.5 and 2 for direct and indirect transitions, respectively). In the Kubelka–Munk approximation, *F*(*R*) is taken as proportional to the absorption coefficient (α), and is therefore used in place of α in the Tauc plot. Afterward, the band gap is obtained from the intersection of the linear fit with the abscissa axis in the (α*h*ν)^1/*n*^ vs *h*ν plot.^50^ The *E*_g_ values obtained were 3.38 and 2.99 eV (367 and 415 nm) for the direct and indirect transitions (see Figure S20). Consequently, light sources emitting in the near-UV and visible violet region could a priori be suitable to promote the generation of photocharges in the Ti_8_BTC metal−organic polymer to initiate complementary reduction and oxidation reactions (see further details in Section 3.6).

**Figure 11:**
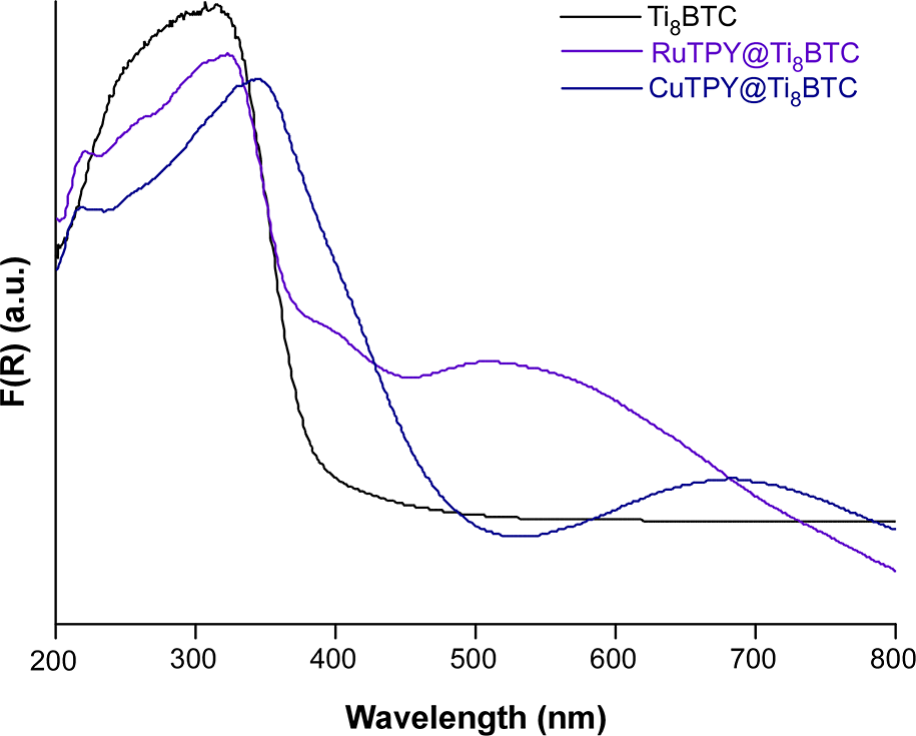
UV–Vis absorption spectra derived from the Kubelka–Munk function *F*(*R*) for Ti_8_BTC, RuTPY@Ti_8_BTC and CuTPY@Ti_8_BTC.

On the other hand, the samples doped with RuCl_3_ and RuTPY complexes show an additional band around 400–420 nm ascribable to ligand-to-metal charge transfer (LMCT; π­(L) → dπ­(M)), which overlaps as a shoulder with that of the Ti_8_BTC and extends notably toward longer wavelengths, obscuring weaker metal centered d–d transitions.^51,52^ In the case of RuBPY@Ti_8_BTC and RuPHEN@Ti_8_BTC, they exhibit higher absorption in the 400–600 nm region, indicating the presence of more pronounced LMCT transitions.^53^ In particular, RuBPY@Ti_8_BTC shows a broad band extending up to 700 nm, which could be attributed to greater π conjugation and electronic coupling between the metal and the ligand.^54^

The inclusion of copper in CuTPY@Ti_8_BTC results in a slight broadening of the first band (related dπ­(M) → π*­(L) type metal-to-ligand charge transfer; MLCT), in addition to the presence of a new band in the visible range with peak maximum at 690 nm ascribed to d–d transitions.^55,56^ It is observed that CoTPY@Ti_8_BTC presents a similar spectrum to CuTPY@Ti_8_BTC, with an intense band around 330–350 nm and an extension toward the visible, which could be associated with MLCT transitions and typical d–d transitions of cobalt in distorted octahedral environments.^57^ On the other hand, NiTPY@Ti_8_BTC shows a less intense absorption band in the UV region, but with a slight extension into the visible range, suggesting a lower efficiency in the generation of excited states.

In addition, we also recorded the photoluminescence (PL) spectra of the pristine, Ru- and Cu-doped samples in comparison to the H_3_BTC organic ligand employed as linker under excitation at 365 nm, corresponding to the wavelength employed during the photocatalytic experiments (Figure S21). The free linker (H_3_BTC) exhibits a broad emission centered around 430 nm, which is almost completely quenched upon coordination with titanium oxoclusters to form the Ti_8_BTC polymer. This strong quenching indicates that radiative recombination of photoexcited carriers is already largely suppressed in the pristine aerogel, a favorable feature for photocatalysis, as it suggests efficient nonradiative charge separation pathways.

### 3.6 Photocatalytic Hydrogen Production

Photocatalytic H_2_ production experiments were performed using prepared MOAs as catalysts and an aqueous solution containing as sacrificial electron donor 0.1 M of triethanolamine (TEOA) at pH = 7.0. According to the absorption edge of Ti_8_BTC, near UV LED light (365 nm) was selected as irradiation source, while no traces of hydrogen production was detected under visible light illumination (405 and 450 nm). It deserves to note that the energy of the valence band upper edge (VBE) for Ti_8_BTC was determined by XPS measurements in the low binding energy range (Figure 12a). This level is 2.83 eV below the Fermi level of the spectrometer, which is 4.447 eV according to the instrument supplier. Therefore, the energy value referred to the vacuum is 7.28 eV and translated into NHE potential is +2.84 V (*E*(VBE, NHE) = *E*(VBE, vacuum) – 4.44 eV).^58,59^ Considering the direct band gap value of Ti_8_BTC (3.38 eV), the position of the conduction band lower edge (CBE), is estimated to be −0.54 V. Thus, the band alignment of Ti_8_BTC provides suitable potentials to drive both half-reactions involved in the photocatalysis experiment (Figure 12b). TEOA/TEOA^+^ half-reaction potential was retrieved from the literature.^60^

**Figure 12:**
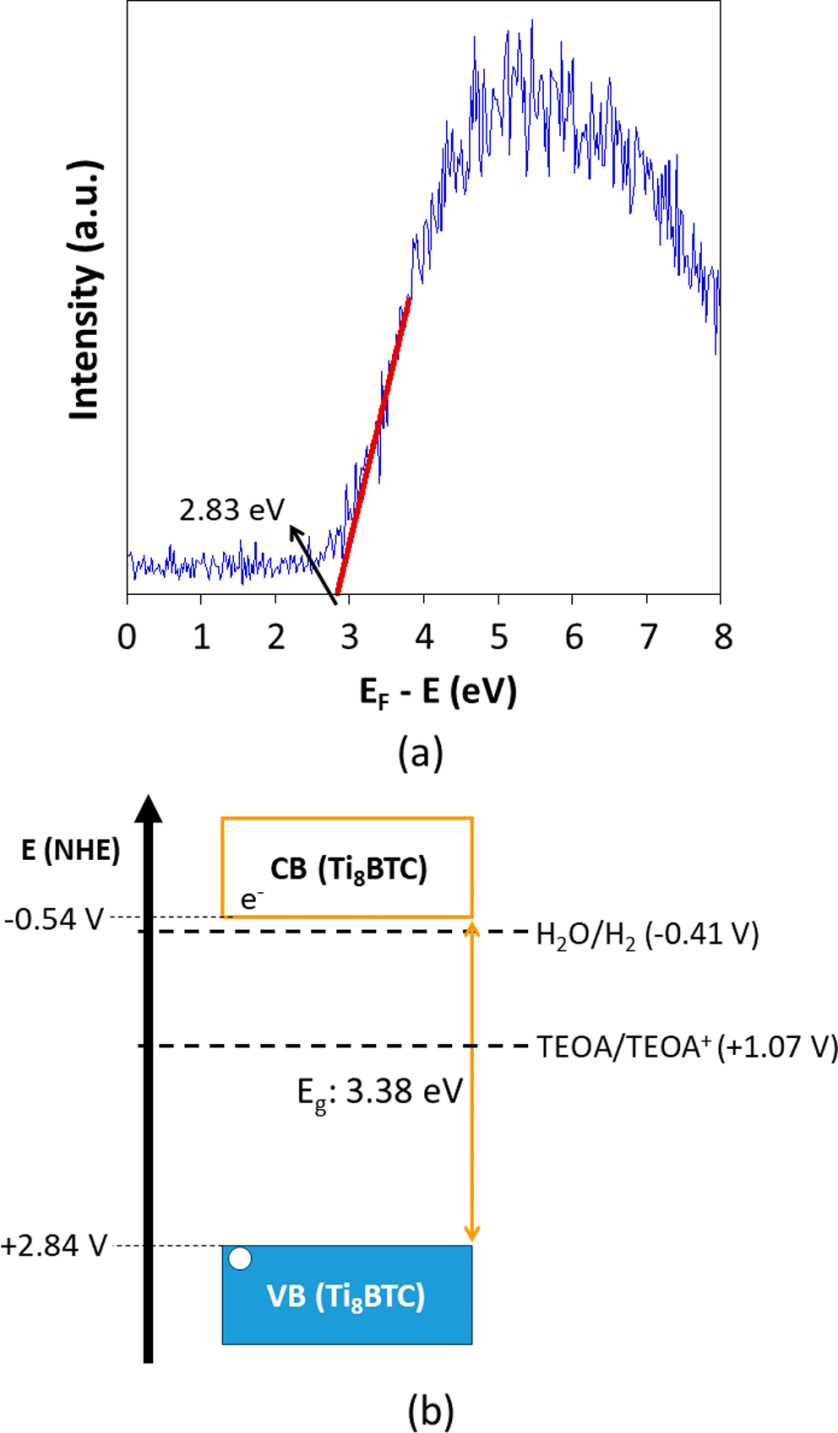
(a) XPS spectrum of the low binding energy region of Ti_8_BTC. (b) Band alignment for Ti_8_BTC compared to the potential of water reduction and TEOA oxidation half-reactions at pH = 7. The band alignment of Ti_8_BTC was determined combining the low binding energy XPS and DRS data.

For comparative purposes, initial experiments were conducted over a duration of 2 h to evaluate the HER performance of Ti_8_BTC (neat and in the presence of Pt nanoparticles) and of Ti_8_BTC doped with the selected metal complexes. Subsequently, long-term hydrogen production was analyzed for both the parent MOA and the best-performing doped MOA. Figure 13 depicts the influence of the amount of Ti_8_BTC in the hydrogen production. The obtained data indicate that, although a greater amount of catalyst favors a higher total yield of hydrogen production (73, 114, and 174 μmol·h^–1^ for Ti_8_BTC catalyst loadings of 625, 1250, and 2500 mg·L^–1^, respectively), it does not translate into a proportional improvement in catalytic activity. In fact, the maximum activity is achieved for the lowest catalyst concentration (110.87 μmol·g^–1^·h^–1^). This phenomenon could be attributed to factors such as agglomeration of catalyst particles, decreased light absorption efficiency, or mass transfer limitations, which reduce the accessibility of the active sites when a higher amount of catalyst is used. Furthermore, it is observed that the error bars are more pronounced for higher Ti_8_BTC concentrations. This greater variability in the results could be attributed to the favored random formation of dead cores (regions of the catalyst that are inaccessible to the reactant) as the catalyst amount increases.^61,62^ As a consequence, a catalyst concentration of 625 mg·L^–1^ (i.e., 5 mg of catalyst under the reaction conditions) was set as optimal for subsequent experiments.

**Figure 13:**
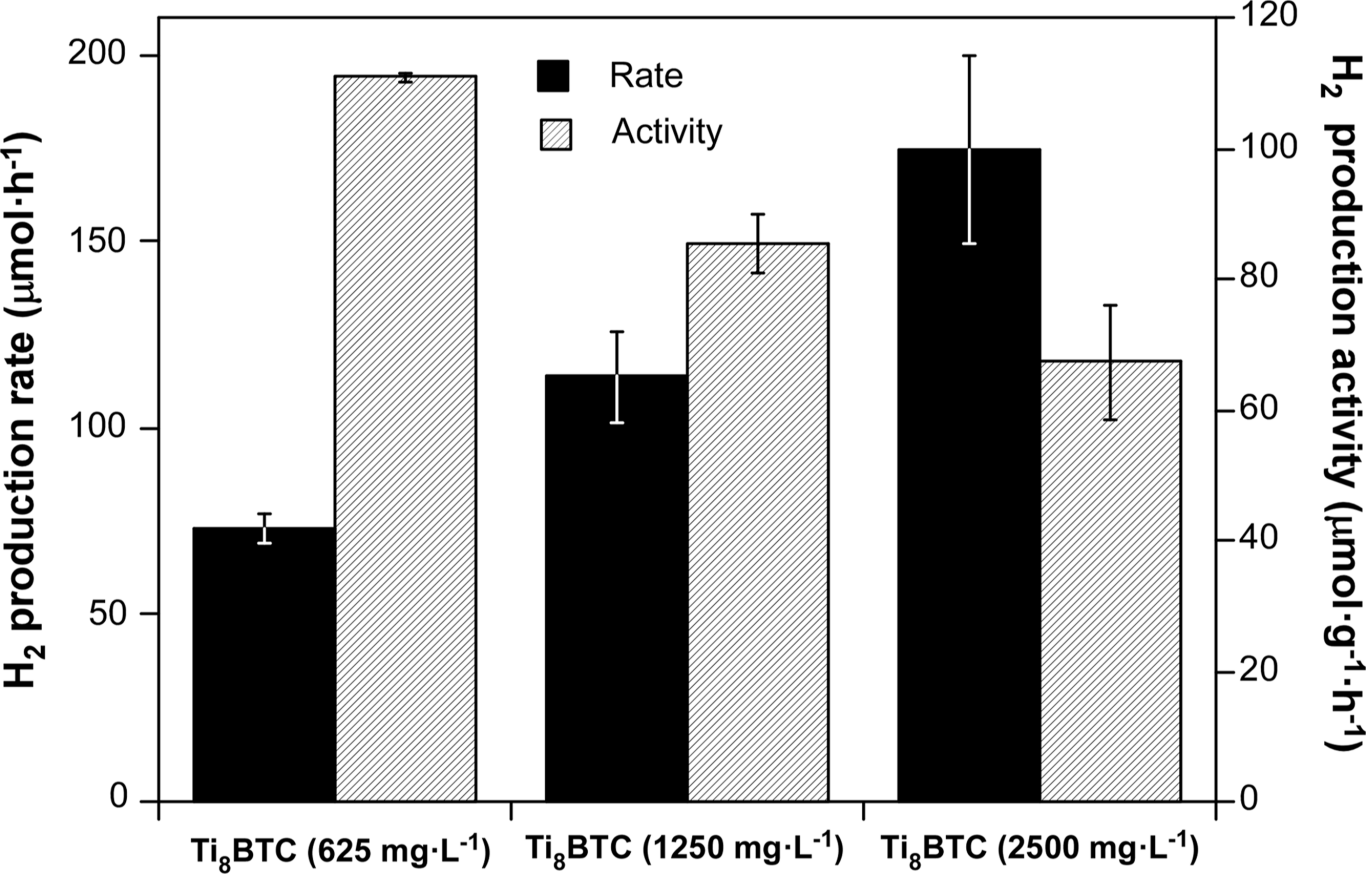
Hydrogen production rate and HER activity for varying Ti_8_BTC concentrations (*C*_1_: 625 mg·L^–1^; *C*_2_: 1250 mg·L^–1^; *C*_3_: 2500 mg·L^–1^) after 2 h of reaction.

As previously mentioned, the photocatalytic mechanism in this class of metal−organic assemblies typically involves the excitation of an electron from the HOMO, which is primarily composed of π-type orbitals of the ligand, to the LUMO, consisting of vacant d orbitals of the metal.^10^ It should be noted that although MOFs are crystalline materials with band-like electronic structures, here the energy levels are discussed in terms of the molecular orbitals that dominate their electronic behavior, for conceptual clarity. Accordingly, the analysis of the frontier orbitals computed via DFT calculations for a structural model of the octanuclear Ti_8_O_8_ cluster indicates that the HOMO and LUMO levels are predominantly composed of the π-symmetry orbitals of the aromatic carboxylato ligands and the unoccupied d orbitals (t_2g_ symmetry) of the titanium metal centers, respectively.^63^ It is worth noting that the UV absorption of the formate ligand^64^ and the benzoate ligand (absorption edge ca. 300–310 nm, Figure S19b) lies below the excitation wavelength used in the experiments (365 nm), and thus they are not expected to contribute significantly to photocharge generation. In contrast, the BTC ligand shows a shifted absorption edge toward higher wavelengths, dominating the first absorption band of Ti_8_BTC (360–380 nm), ascribable to π–π transitions. This assignment is further supported by photocatalytic HER experiments performed with Ti_8_BNZ, a precursor containing only benzoate ligands, which yielded no measurable hydrogen. These results indicate that is the BTC ligand the one which plays a key role in the initial generation of photocharges under UV illumination. The electron transition, which may occur through intersystem conversion (ISC) or ligand-to-metal charge transfer (LMCT), results in the spatial separation of photoinduced charges (holes, h^+^, and electrons, e^–^). This charge separation increases the average lifetime of the charges, facilitating the corresponding oxidation and reduction photoreactions. As a result, there is a reduction of Ti­(IV) to Ti­(III), which herein was evidenced by a visible color change in the sample, attributed to light absorption-induced d–d transitions in octahedral titanium­(III) complexes (t_2g_^1^ e_g_^0^), which typically produce a blue-violet coloration. Previous studies in Ti MOFs have also reported this behavior and confirmed this process by electron paramagnetic resonance (EPR) studies.^65,66^ Once the reaction concluded, the blue coloration of Ti_8_BTC gradually fades as the transient Ti­(III) species are oxidized back to Ti­(IV). This observation suggests that the photoreduction of water (i.e., hydrogen evolution reaction, HER) has a slower kinetic than the complementary photooxidation of TEOA.

Considering the above-mentioned, the HER performance of Ti_8_BTC was evaluated using varying amounts of Pt nanoparticles (0.5, 1, and 2%, w/w), which are commonly employed as benchmark co-catalysts in photocatalytic hydrogen production due to their proven ability to enhance the transfer of photoelectrons, thereby significantly accelerating the reaction rates.^67^ Compared to the neat catalyst, the photocatalytic activity increases significantly using 1% of Pt NPs (150 μmol·g^–1^·h^–1^ vs 110 μmol·g^–1^·h^–1^ for neat Ti_8_BTC). By increasing the Pt concentration to 2% a decrease in activity (117 μmol·g^–1^·h^–1^) is observed compared to 1%, although it is still slightly higher than the catalyst without Pt. This reduction in activity could be related to co-catalyst oversaturation, which could cause shadowing effects or blocking of active sites on the catalyst. The use of 0.5% Pt shows comparable activity (99 μmol·g^–1^·h^–1^) to that of the platinum-free catalyst, indicating that a minimum threshold of Pt concentration needs to be exceeded to ensure an effective contact with the Ti_8_BTC catalyst. It deserves to note that in the presence of the co-catalyst the MOA retains its pristine color during the reaction, since platinum speeds up the transfer of the photoelectrons and inhibit their accumulation in the titanium-oxo cluster.

Despite the widespread use of platinum to promote hydrogen production, this approach to utilize the co-catalyst involves a certain degree of inefficiency in the use of the metal, which is somewhat mitigated by increasing the surface-to-volume ratio when reducing the particles to nanoscale sizes.^68,69^ On the contrary, single-atom catalyst systems, which contain singly dispersed metal atoms on supports, maximize the exploitation of used metal, implying a meaningful advantage for precious metal-based catalyst systems.^70^ In this sense, the herein reported post-synthetic metalation of Ti_8_BTC allowed to get dispersed single-atom sites by the chemical anchoring of the co-catalyst complexes at the free carboxylic ligands of the coordination framework. Precisely, we selected a series of ruthenium species since this metal has been pointed out as a promising alternative to platinum, due to its low cost and moderate metal–hydrogen bond strength (close to Pt–H).^71,72^ The highest HER activity values obtained in the photocatalytic experiments corresponded to the MOA functionalized with ruthenium (RuCl_3_@Ti_8_BTC: 124 μmol·g^–1^·h^–1^) and ruthenium/terpyridine complex (RuTPY@Ti_8_BTC: 167 μmol·g^–1^·h^–1^), which surpassed the undoped catalyst. Note that the activity of RuTPY@Ti_8_BTC outranges also that provided by Ti_8_BTC in the presence of Pt nanoparticles (1.0 wt %), even though the amount of Ru introduced was lower (0.41 wt %). The superior performance of RuTPY@Ti_8_BTC can be attributed to the ability of metal-terpyridine complexes to store multiple reducing equivalents, which arises from the capability of the nitrogen ligands to stabilize reduced states of metal center favoring the transfer of the photoelectron to the hydrogen evolution reaction.^73,74^ However, MOA doped with ruthenium complexes of bidentated N-ligands (2,2′-bipyrydine and 1,10-phenanthroline) exhibited lower photocatalytic activity than the neat photacatalyst (49 and 33 μmol·g^–1^·h^–1^ for RuBPY@Ti_8_BTC and RuPHEN@Ti_8_BTC, respectively). These results suggest that although nitrogen-containing ligands, such as terpyridine, can improve electron transfer to some extent, the phenanthroline and bipyridine complexes are less efficient. This behavior could be attributed to both the lower number of donor nitrogen atoms involved in the complex, which might reduce their ability to stabilize reactive intermediates and facilitate efficient charge transfer, and the light absorption of the Ru/ligand complexes, which competes with generation of photocharges at Ti_8_BTC.

Motivated by the superior results obtained with Ru/terpyridine doped aerogels, where the terpyridine ligand played a critical role in enhancing activity, we decided to explore the performance of the Ti_8_BTC MOA functionalized with terpyridine complexes of rather cheaper metals (cobalt, nickel, and copper), which have been investigated as alternative HER catalysts to platinum group metals (PGM).^72^ The photocatalytic performance of the resulting materials was evaluated under identical experimental conditions to those used for RuTPY@Ti_8_BTC. The results of the hydrogen production are gathered in Figure 14. Contrary to expectations, NiTPY@Ti_8_BTC displays significantly low hydrogen production (83 μmol·g^–1^·h^–1^), while CoTPY@Ti_8_BTC (105 μmol·g^–1^·h^–1^) does not get over the performance of the neat MOA. Interestingly, the hydrogen production activity of CuTPY@Ti_8_BTC (164 μmol·g^–1^·h^–1^) equals that of ruthenium analogous (RuTPY@Ti_8_BTC) and it gets over the performance of Ti_8_BTC when using Pt NP as co-catalyst. Considering the functionalization with metal/terpyridine complexes, the better performance provided by ruthenium and copper can be probably related to their positive standard reduction potentials (or noble metal character) that eases getting reduced states by incoming photoelectrons and their subsequent transfer to HER. Control experiments using the neat RuTPY and CuTPY complexes did not produce any measurable hydrogen. This observation, together with the fact that inclusion of CoTPY and NiTPY complexes reduces HER activity compared to the pristine Ti_8_BTC, supports the idea that the enhanced performance observed with Ru and Cu dopants may arise from improved photophysical processes, such as charge separation and transfer, although further studies are needed to establish the exact mechanism.

**Figure 14:**
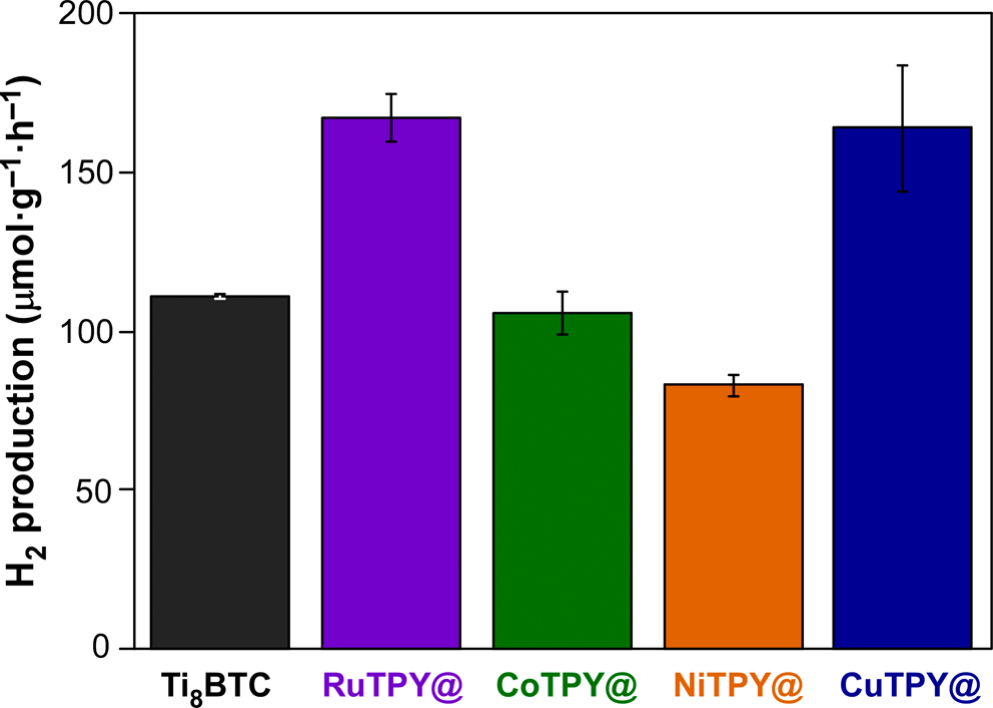
HER activity for Ti_8_BTC doped with metal complexes of terpyridine. The graph includes the data of neat Ti_8_BTC for comparative purposes. In all cases the catalyst concentration was set to 625 mg·L^–1^.

Finally, to evaluate the stability of the catalytic system, experiments were conducted over a period of 24 h with the undoped aerogel (Ti_8_BTC) and the aerogels doped with RuTPY and CuTPY complexes, which demonstrated one of the best performances in the previous tests. The results of these experiments are shown in Figure 15 (see numerical data with associated errors in Table S7). The undoped aerogel shows an initial increase in the production rate to reach a maximum of 109 μmol·g^–1^·h^–1^ after 150 min of operation. Such initial activity increase can be related to the limited capability of the titanium oxo-clusters to conduct the reduction of hydrogen, that initially acts as drain of photoelectrons by means of the eventual reduction to Ti­(III) upon illumination. However, the functionalization with RuTPY and CuTPY eases the transfer of the photoelectron and speeds up the HER rate to reach a maximum of 179 μmol·g^–1^·h^–1^ and 170 μmol·g^–1^·h^–1^ at a shorter reaction time (both of them in 120 min). Thereafter, all MOAs exhibit a gradual decline in the catalytic activity over time, reaching roughly steady state after 12 h of operation, with activity values of 104, 155, and 143 μmol·h^–1^·g^–1^, for Ti_8_BTC, RuTPY@Ti_8_BTC and CuTPY@Ti_8_BTC respectively.

**Figure 15:**
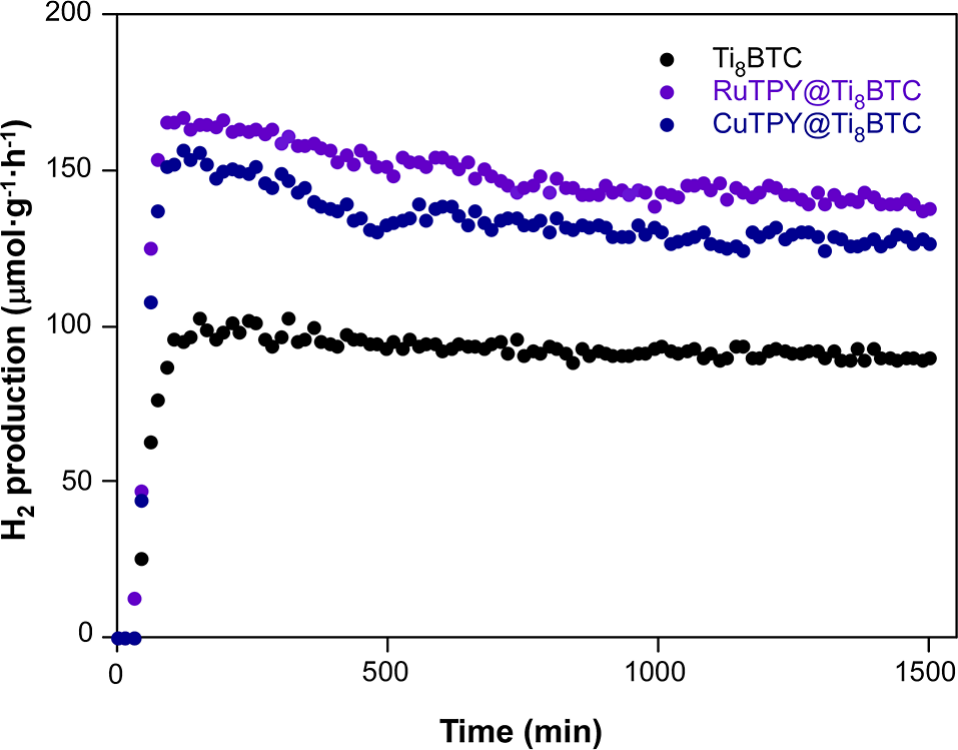
Evolution HER activity during 24 h of reaction for Ti_8_BTC, RuTPY@Ti_8_BTC and CuTPY@Ti_8_BTC.

## 4 Conclusions

The two-step synthesis procedure, involving the preformation of a Ti_8_ oxo-cluster followed by the subsequent exchange of terminal ligands with benzene-1,3,5-tricarboxylato (BTC) linkers demonstrated to be a successful and more controlled route for preparing titanium-based metal−organic aerogels (MOAs). Thoughtful chemical analysis, NEXAFS spectroscopy and HAADF−TEM analysis confirmed the structural retention of the Ti_8_ clusters within the resulting noncrystalline porous MOA, which exhibited a hierarchical meso-/macroporosity as evidenced by N_2_ sorption isotherms. Furthermore, the cross-linking among the selected building blocks rendered free carboxylic groups available at the pore surface of the Ti_8_BTC MOA. These functional groups provided key sites for the post-synthetic functionalization of Ti_8_BTC with co-catalyst species containing terpyridine (TPY), bipyridine (BPY) and phenanthroline (PHEN) complexes. As a result, singly dispersed metal complexes of ruthenium, nickel, cobalt, and copper were anchored onto the metal−organic surface, as evidenced by XPS and EDX elemental mapping. Notably, compared to the use of co-catalyst nanoparticles, this approach allows for more efficient utilization of the metal species.

Photocatalytic hydrogen evolution experiments under UV irradiation revealed that only the materials functionalized with terpyridine complexes of ruthenium (RuTPY) and copper (CuTPY) displayed a marked enhancement in activity compared to both the pristine Ti_8_BTC aerogel and the one adding Pt nanoparticles as co-catalyst. The improved performance of RuTPY@Ti_8_BTC and CuTPY@Ti_8_BTC may be attributed to the extended π-conjugation and chelating ability of the terpyridine ligands, which facilitate efficient electronic communication and photoinduced charge separation within the framework. In contrast, the NiTPY and CoTPY functionalized materials displayed negligible improvement, likely due to the insufficient alignment between their electronic orbitals and the conduction band of Ti-based clusters. Similarly, complexes containing RuBPY and RuPHEN complexes did not lead to notable improvements in photocatalytic activity. This difference may arise from the coordination geometry and electronic properties of these ligands, which are less extended and conjugated than terpyridine, resulting in less efficient charge delocalization and weaker interaction with the Ti_8_BTC scaffold. The material doped with RuCl_3_, although active, showed lower performance than RuTPY@Ti_8_BTC, possibly due to the absence of a stabilizing ligand environment capable of promoting charge delocalization.

These results demonstrate that the photocatalytic behavior of Ti_8_BTC aerogels can be finely tuned through the choice of post-synthetically coordinated metal complexes, and that both the nature of the metal center and the structure of the ligand play a crucial role in determining activity. In particular, RuTPY- and CuTPY-modified materials offer a promising alternative to noble metal nanoparticles for hydrogen evolution, combining high efficiency with molecular-level control over composition and active site distribution.

## Supplementary Material



## Notes

Experimental procedures for the synthesis of Ru-, Co-, Ni- and Cu-based metal complexes; PXRD, TGA, and CHN elemental analysis data of the metal complexes; additional NMR, TGA, FTIR, PXRD, TEM, SEM, N_2_ adsorption–desorption isotherms, pore size distribution, XPS, UV–Vis DRS and PL data of MOAs; and numerical data with error analysis of photocatalytic hydrogen evolution experiments (PDF)
