# Supplementary material for: Cluster-Engineered Titanium Metal−Organic Aerogels as Tunable Platforms for Post-synthetic Doping and Enhanced Photocatalytic Hydrogen Production
Source: Inorg Chem. 2025 Dec 15;64(51):25089–104. doi: 10.1021/acs.inorgchem.5c03886 (PMC12754791; doi:10.1021/acs.inorgchem.5c03886)
Supplement: Supplementary file 1 [file ic5c03886_si_001.pdf]

Supporting Information for

Cluster-engineered titanium metal-organic aerogels as tunable platforms for post-synthetic doping and enhanced photocatalytic hydrogen production

Naia Luengo,[a] Ane Ciruela-Zunzunegui,[a,b] Maite Perfecto-Irigaray,[a,c]* Oscar Castillo,[a,d] Pilar Ferrer,[e] Matthijs A. van Spronsen,[e] Sonia Pérez-Yáñez[a,d] and Garikoitz Beobide[a,d]*

[a] Department of Organic and Inorganic Chemistry, Faculty of Science and Technology, University of the Basque Country (UPV/EHU), 48940 Leioa, Spain.

[b] Department of Chemistry, Università degli Studi di Milano, Via Golgi 19, 20133 Milan, Italy.

[c] ISIS Neutron and Muon Source, STFC Rutherford Appleton Laboratory, Didcot OX11 0QX, U.K.

[d] BCMaterials, Basque Center for Materials, Applications and Nanostructures, UPV/EHU Science Park, Leioa 48940, Spain.

[e] Diamond Light Source, Harwell Science and Innovation Campus, Didcot OX11 0DE, U.K.

*Contact email: maite.perfecto@ehu.eus; garikoitz.beobide@ehu.eus.

S1. SYNTHESIS OF THE METAL COMPLEXES S2
S2. CHARACTERIZATION OF THE METAL COMPLEXES S4
S2.1. Powder X-Ray diffraction (PXRD) S4
S2.2. Thermogravimetric analysis (TGA) S5
S2.3. Elemental analysis (CHN) S5
S3. CHARACTERIZATION OF MOAs S6
S3.1. Proton nuclear magnetic resonance (^{1}H-NMR) S6
S3.2. Thermogravimetric analysis (TGA) S7
S3.3. Fourier-transform infrared spectroscopy (FTIR) S9
Quantification of accesible –COOH groups: S10
S3.4. Powder X-ray diffraction (PXRD) S11
S3.5. Transmission electron microscopy (TEM) S12
S3.6. Scanning electron microscopy (SEM) S13
S3.7. N_2 adsorption isotherms and cumulative pore size distribution S14
S3.8. X-ray photoelectron spectroscopy (XPS) S15
S3.9. UV-Vis diffuse reflectance spectroscopy (DRS) S18
S3.10. Photoluminescence (PL) measurements S18
S4. PHOTOCATALYTIC HYDROGEN PRODUCTION S20
S4.1. Hydrogen evolution experiments S20
S4.2. Apparent quantum efficiency (AQE) calculations S22

S1. SYNTHESIS OF THE METAL COMPLEXES

This section describes the synthesis of the different metal complexes used in the metalation studies. The synthesis of each complex was adapted from previously reported procedures.[1,2,3] The molecular structures of the obtained compounds are shown in Fig. S1.

Synthesis of [$RuCl_3$(TPY)] (RuTPY). In a 200 mL round-bottom flask containing absolute ethanol (125 mL) $RuCl_3·3H_2O$ (0.26 g, 1.00 mmol) and 2,2':6',2''-terpyridine (0.23 g, 1.00 mmol) were added. Then, the reaction mixture was heated up at reflux for 3 h, while vigorous magnetic stirring was maintained. Afterwards, the reaction was cooled down to room temperature, and the resulting brown powder was filtered. The final product was washed in triplicate with 30 mL of absolute ethanol and dried in an oven at 80 °C overnight. The compound was obtained as a brown powder with 87.6% yield.

Synthesis of [$RuCl_2$(BPY)(OH_2)$_2$]Cl (RuBPY) and [$RuCl_2$(PHEN)(OH_2)$_2$]Cl (RuPHEN). $RuCl_3·3H_2O$ (1.57 g, 6.00 mmol) and 2,2'-bipyridine (1.87 g, 12.00 mmol) or 1,10-phenanthroline (2.16 g, 12.00 mmol) were combined in 30 mL of absolute ethanol and heated at reflux for 12 h while magnetic stirring was maintained. After this time, the reaction mixture was allowed to cool down to room temperature, and the obtained brown solid was filtered. The crude product was added to 700 mL of a very hot solution of NaCl (40 g) in H_2O and allowed to stir for 15 min. The solution was filtered hot, and the filtrate volume was reduced to one-third on a rotary evaporator, yielding, in both cases, brownish orange powder. The product was isolated by vacuum filtration, washed with a small portion of cold H_2O, and kept in a vacuum desiccator overnight (yield: 54% and 61.5%, respectively).

Synthesis of [Co(TPY)Cl_2] (CoTPY) and [Cu(TPY)Cl_2] (CuTPY). A methanolic solution (20 mL) of 2,2':6',2''-terpyridine (0.23 g, 1.00 mmol) was added dropwise to a methanolic solution (10 mL) of $CoCl_2·6H_2O$ (0.24 g, 1.00 mmol) or $CuCl_2·6H_2O$ (0.24 g, 1.00 mmol) with constant stirring for 2 hours. Then the coloured solution was filtered and the filtrate was kept undisturbed for slow evaporation. After one week, blue-green crystals for CoTPY or green crystals for CuTPY were obtained (yield: 65% and 61.5%, respectively).

Synthesis of [Ni(TPY)Cl(OH_2)$_2$]Cl (NiTPY). A methanolic solution (10 mL) of 2,2':6',2''-terpyridine (0.23 g, 1.00 mmol) was added dropwise to a methanolic solution (10 mL) of $NiCl_2·6H_2O$ (0.24 g, 1.00 mmol) in the with constant stirring which continued for 3 hours. Then the solution was filtered and the filtrate was left for slow evaporation. After one week, deep green crystals were isolated (yield: 62%).

[1] Y. Cheret, A. Szukalski, K. A. Haupa, A. Popczyk, J. Mysliwiec, B. Sahraoui and A. El-Ghayoury, *Polyhedron*, 2023, **233**, 116299.

[2] P. Pal, K. Das, A. Hossain, A. Frontera and S. Mukhopadhyay, *New J. Chem.*, 2020, **44**, 7310–7318.

[3] D. S. Eggleston, K. A. Goldsby, D. J. Hodgson, T. J. Meyer, *Inorg. Chem.* 1985, **24**, 4573–4580.

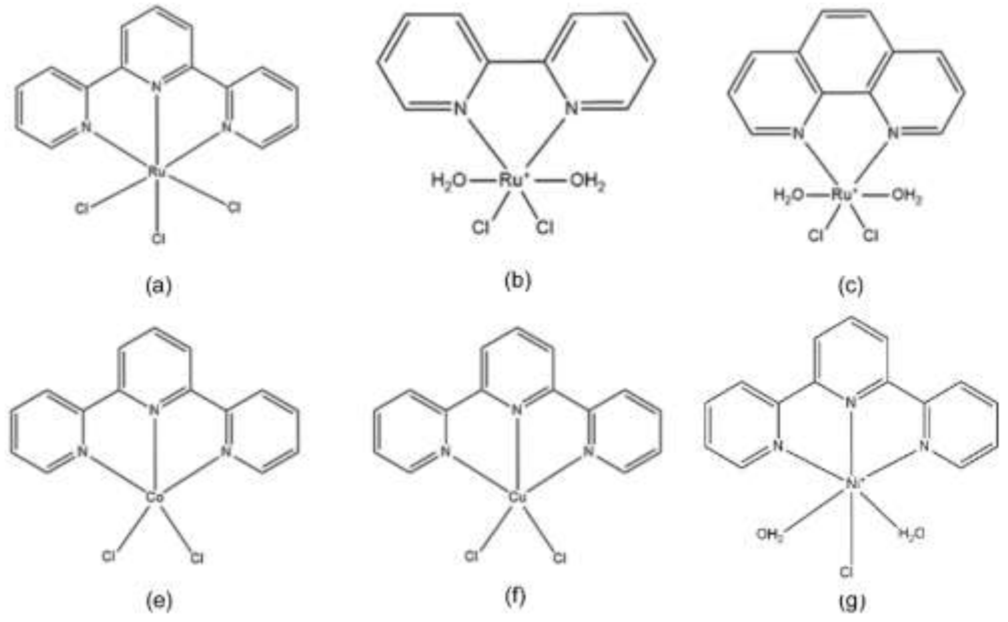

Fig. S1 Structures of the metal complexes used for metalation studies: (a) RuTPY, (b) RuBPY, (c) RuPHEN, (d) CoTPY, (e) CuTPY and (f) NiTPY.

S2. CHARACTERIZATION OF THE METAL COMPLEXES

S2.1. Powder X-Ray diffraction (PXRD)

The phase purity and structural integrity of the prepared metal complexes were analysed by PXRD (Fig. S2). The diffractograms show that the prepared complexes match well with the theoretical patterns found in literature[4,5,6,7], confirming the expected crystalline structure. However, for the newly synthesized RuBPY and RuPHEN complexes, no specific reference PXRD patterns were available in the literature. As a result, their experimental formulas were deduced from thermogravimetric (TG) and elemental analyses, as presented below.

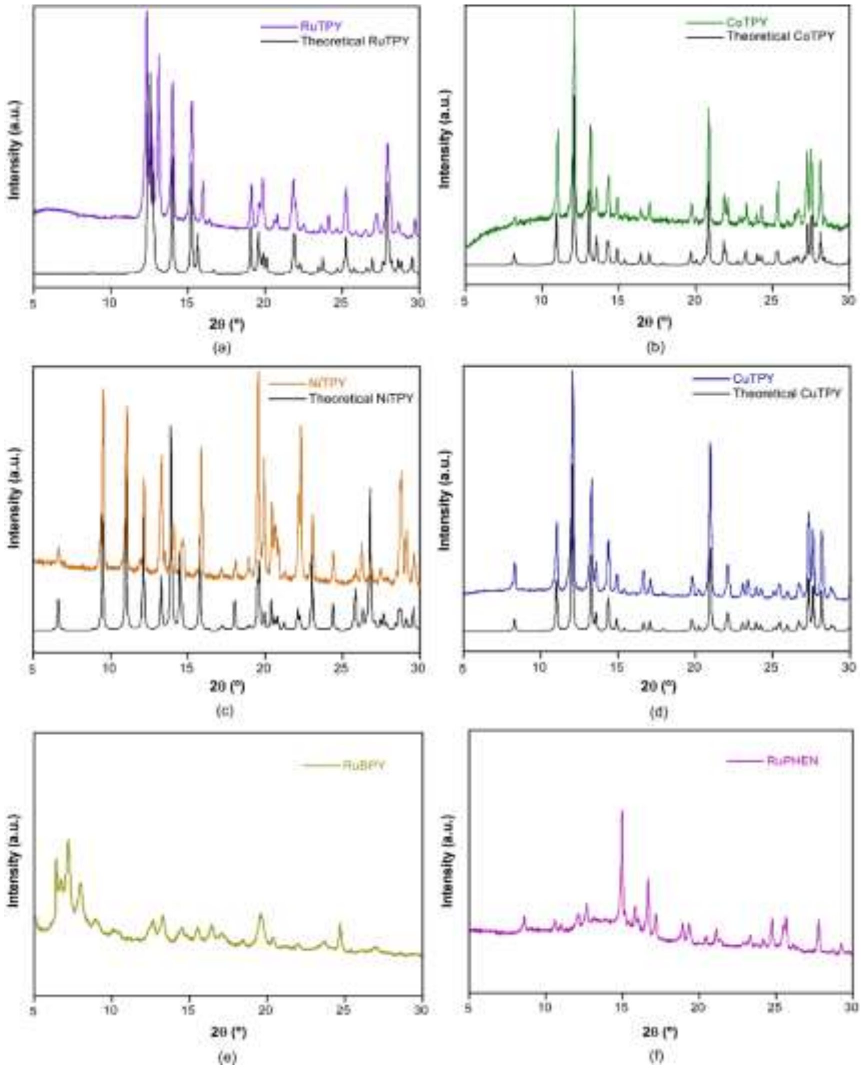

Fig. S2 PXRD of (a) RuTPY, (b) CoTPY, (c) NiTPY, and (d) CuTPY in comparison with theoretical ones retrieved from the Cambridge Structural Database (CUBQEV, CTPYCO, DESKAN and ZZZKWM entries, respectively)[8] and PXRD of new phases of (e) RuBPY and (f) RuPHEN.

---

[4] F. Laurent, E. Plantalech, B. Donnadieu, A. Jimenez, F. Hernandez, M. Martinez-Ripoll, M. Biner, A. Llobet, *Polyhedron,* 1999, **18**, 3321–3331.

[5] E. Goldschmied, N. C. Stephenson, *Acta Crystallogr.*, 1970, **26**, 1867–1875.

[6] R. Cortes, M. I. Arriortua, T. Rojo, X. Solans, C. Miravitlles, D. Beltran, *Acta Crystallogr.*, 1985, **41**, 1733–1736.

[7] C. M. Harris, T. N. Lockyer, N. C. Stephenson, *Aust. J. Chem.,* 1966, **19**, 1741–1743.

[8] C. R. Groom, I. J. Bruno, M. P. Lightfoot and S. C. Ward, *Acta Crystallogr. Sect. B Struct. Sci. Cryst. Eng. Mater.*, 2016, **72**, 171–179.

S2.2. Thermogravimetric analysis (TGA)

Thermogravimetric analyses (TGA) were used to determine the thermal stability and to estimate the molecular composition of RuBPY and RuPHEN complexes. The observed weight loss profiles (Fig. S3) allowed the identification of the proportions of the organic and metallic components. Confirming that the final decomposition residue corresponds to RuO_2 (MW = 133.07 g·mol^{-1}), the residual mass fractions (34.16% for RuBPY complex and 28.52% for RuPHEN complex) were used to calculate the experimental molecular weights of the compounds. This yielded values of 382.61 g·mol^{-1} for RuBPY and 426.74 g·mol^{-1} for RuPHEN which are in good agreement with the theoretical values of 399.64 g·mol^{-1} and 423.66 g·mol^{-1} of the proposed molecular formulas: $[RuCl_2(BPY)(OH_2)_2]Cl$ and $[RuCl_2(PHEN)(OH_2)_2]Cl$.

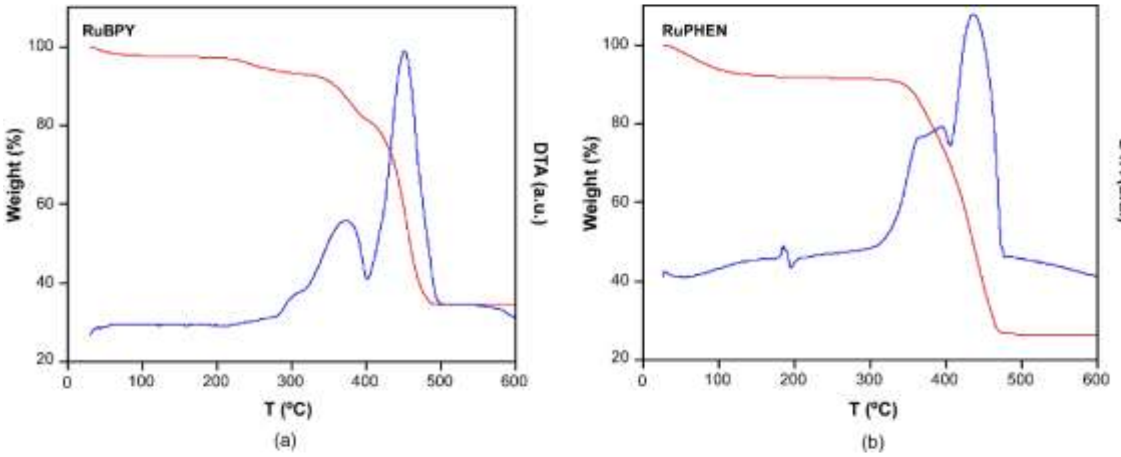

Fig. S3 TGA-DTA curves of RuBPY and RuPHEN complexes.

S2.3. Elemental analysis (CHN)

Elemental analysis (CHN) was performed to verify the composition of RuBPY and RuPHEN complexes. The experimentally obtained values were close to the expected ones, confirming the proposed molecular formula. The slight variations could be attributed to residual solvent molecules or minor structural defects.

Table S1 Comparison of observed and expected CHN elemental analysis values for RuBPY and RuPHEN complexes.

	**Expected**			**Observed**		
**Sample**	**C (%)**	**H (%)**	**N (%)**	**C (%)**	**H (%)**	**N (%)**
**$[RuCl_2(BPY)(OH_2)_2]Cl$**	30.05	3.03	7.01	32.89	2.83	7.76
**$[RuCl_2(PHEN)(OH_2)_2]Cl$**	34.02	2.86	6.61	37.42	3.01	6.89

S3. CHARACTERIZATION OF MOAs

S3.1. Proton nuclear magnetic resonance (^{1}H-NMR)

To proceed with ^{1}H-NMR spectrum (500 MHz), 50 mg of the MOA was digested in 2 mL of a 1 M NaOH solution (in D_2O). The digestion was prolonged for 30 minutes, after which fumaric acid was added as an internal standard (14 mg), and the solid residue was filtered off. The NMR spectrum was then taken on the liquid fraction. Fig. S4 shows the label assignment of the species identified in the NMR spectra of Fig. S5. The singlet at 6.45 ppm is related to the two vinylic ^{1}H-atoms of fumaric acid. The singlet present at 8.32 ppm corresponds to the three aromatic H-atoms of benzene-1,3,5-tricarboxylic acid, while the three chemically distinguishable H-atoms of benzoic acid are featured by three set of signals at 7.78 ppm (doublet), 7.47 ppm (triplet) and 7.39 ppm (triplet). Finally, the singlet at 8.37 ppm observed in all spectra corresponds to the C–H atom of formic acid and the remaining signals (H_E and H_F) are attributed to the solvents used during the synthesis and washing steps.

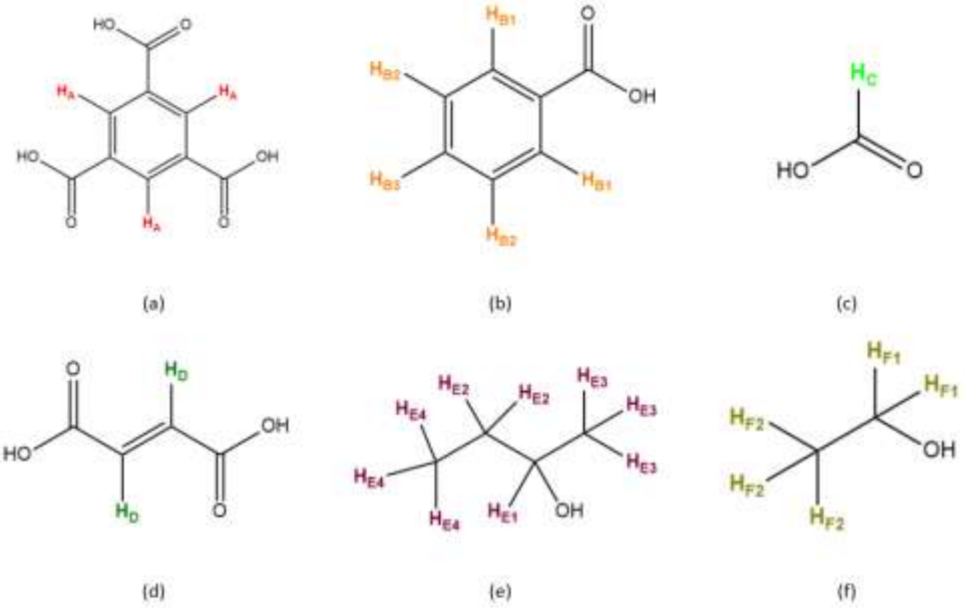

Fig. S4 Proton label assignation for (a) benzene-1,3,5-tricarboxylic acid, (b) benzoic acid, (c) formic acid, (d) fumaric acid, (e) butan-2-ol and (f) ethanol identified in ^{1}H-NMR spectra of Fig. S5.

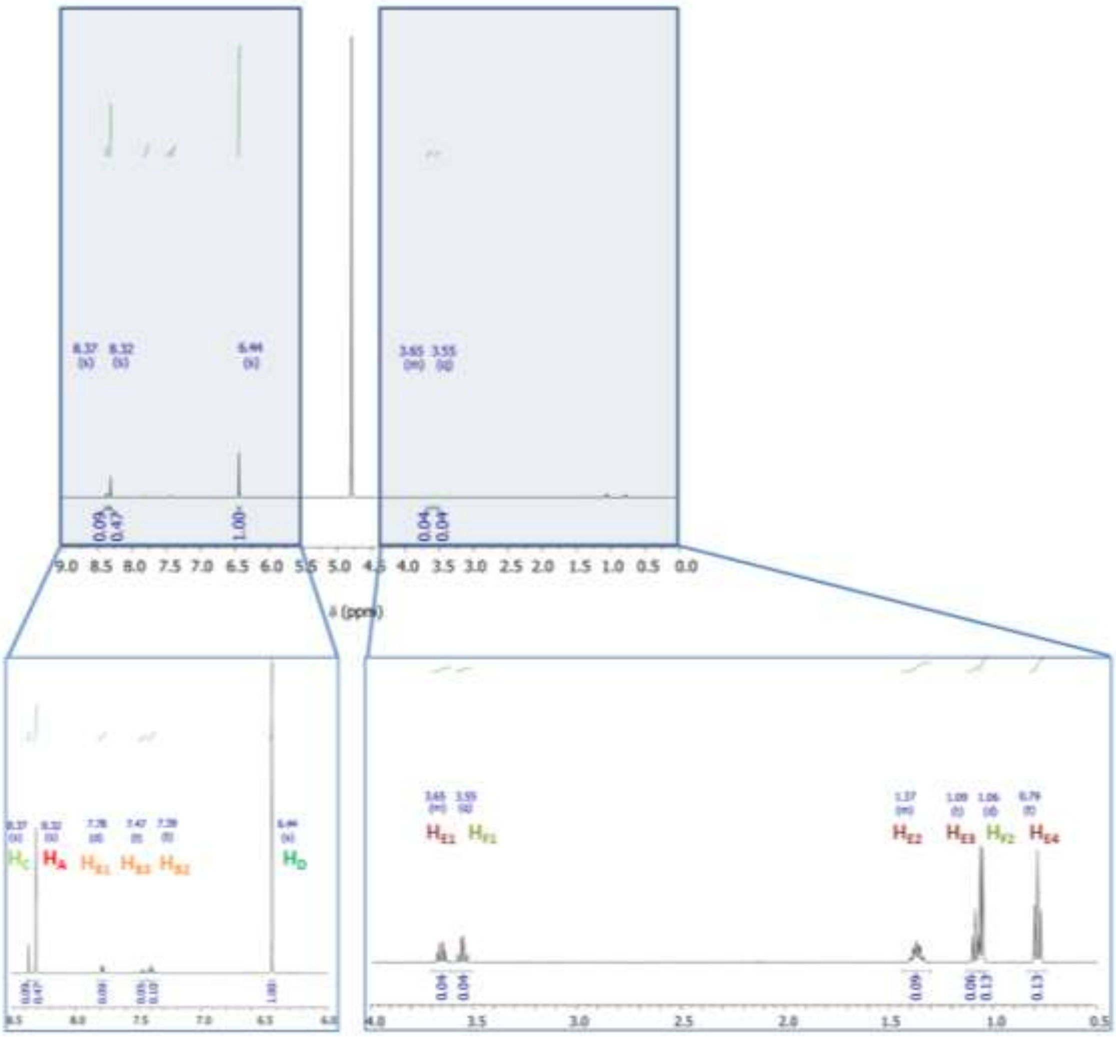

Fig. S5 ^{1}H-NMR spectrum in D_2O of Ti_8BTC.

S3.2. Thermogravimetric analysis (TGA)

The thermogravimetric analysis of MOAs was carried out in synthetic air from 30 °C to 800 °C, revealing three main stages of weight loss (Fig. S6). The first stage, occurring between 30 and 200 °C, shows minimal mass loss, indicating a low amount of solvent trapped within the pores. In the second stage (200 – 400 °C), the release of coordinated water molecules takes place. The most significant weight loss occurs between 400 and 550 °C, corresponding to the decomposition of the framework. An exothermic event around 500 °C suggests the combustion of organic components. In all cases, TiO_2 was formed as final residue, which was identified by PXRD (rutile phase, ICDD PDF No. 00-001-1292).

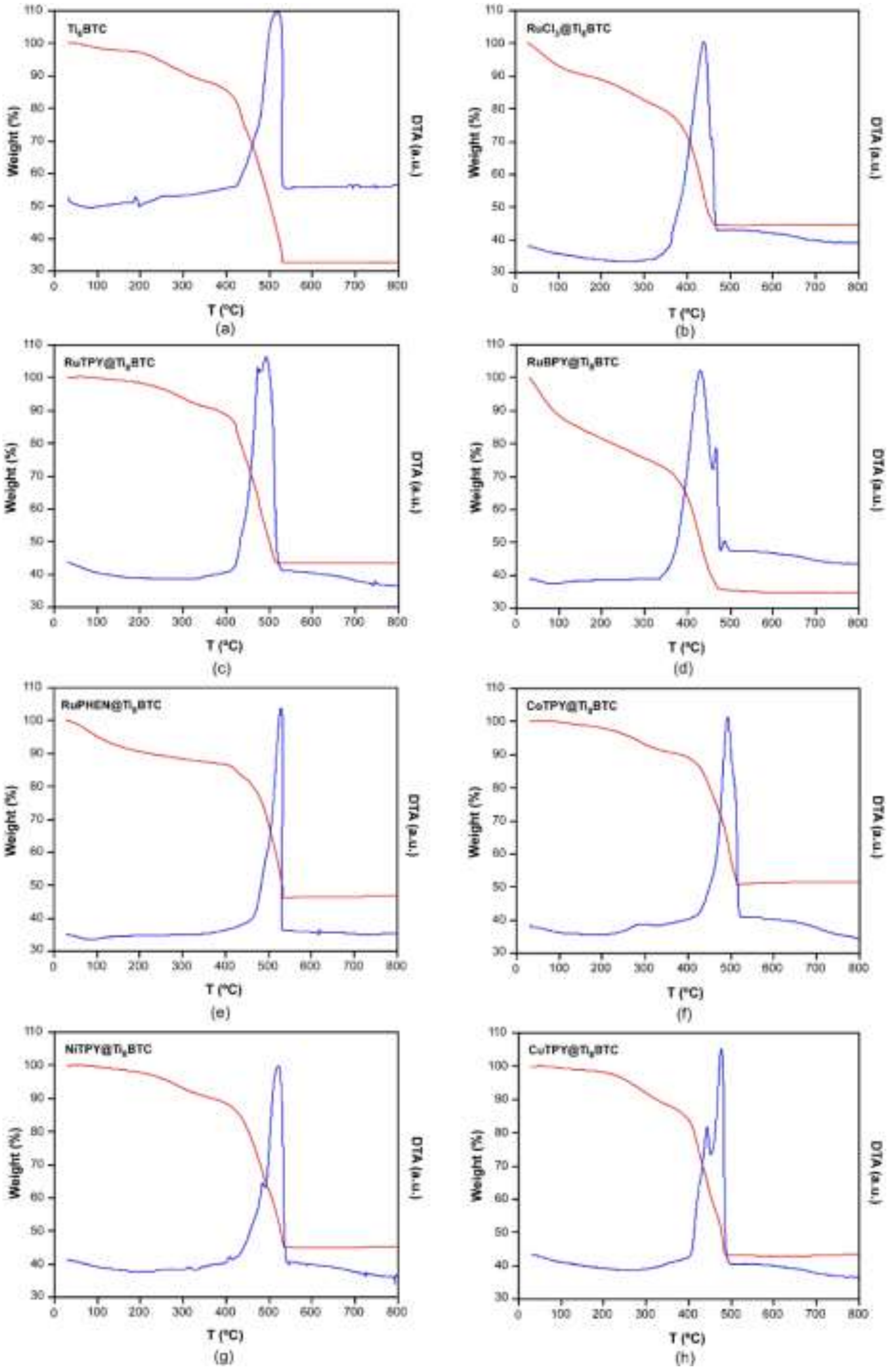

Fig. S6 TGA-DTA curves of (a) Ti_8BTC, (b) $RuCl_3@Ti_8BTC$, (c) RuTPY@Ti_8BTC, (d) RuBPY@Ti_8BTC, (e) RuPHEN@Ti_8BTC (f) CoTPY@Ti_8BTC, (g) NiTPY@Ti_8BTC and (h) CuTPY@Ti_8BTC.

Table S2 compares the estimated formula of Ti_8BTC obtained from elemental analysis with the data derived from TGA. The chemical formula of the product formed in the second step is estimated by assuming that formate release consists of an oxide derived from formate, which also agrees fairly well with the expected formula (1735.3 vs 1704.9 g·mol^{-1}). At low temperatures, according to literature data, formate release can yield hydroxide groups, but at temperatures above 300 °C, these tend to condense into oxide, releasing water.[9] In any case, an alternative formula considering hydroxide involvement,

[9] D. Kristian-Sannes, S. Øien-Ødegaard, E. Aunan, A. Nova and U. Olsbye, *Chem. Mater.*, 2023, **35**, 3793–3800.

$[Ti_8O_8(OH)_{2.84}(BTC)_{4.80}(BNZ)_{1.44}]$, presents a molecular weight that matches the experimental value (1730.5 g·mol^{-1}). It should be noted that the calculated molecular weight (M_F) considers carboxylic protons as estimated in the manuscript for BTC, however, omitting them does not result in significant changes in the overall molar mass.

Table S2 Comparison of TGA data with estimated formulas and molecular weights for Ti_8BTC.[a]

St	T_i	T_f	m_r	Proposed formula	M (g·mol^{-1})		|σ| (%)
					M_{Exp}	M_F	
1	50	150	97.7	$[Ti_8O_8(BTC)_{4.80}(BNZ)_{1.44}(FOR)_{2.84}]\cdot(EtOH)_{0.57}(ButOH)_{1.31}$	1915.6	1933.4	0.92
2	200	350	88.5	$[Ti_8O_{9.42}(BTC)_{4.80}(BNZ)_{1.44}]$	1735.3	1704.9	1.78
3	400	550	32.6	TiO_2	79.9	79.9	0

[a]: St: step. T_i: initial temperature (°C); T_f: final temperature (°C); m_r: residual mass (%) after the decomposition step; M_{Exp} and M_F: experimental (from TGA) and formula-based molecular weights, respectively. σ: deviation (%) between experimental and formula-based molecular weights, calculated as $\sigma = \frac{M_F - M_{Exp}}{M_F} \cdot 100$.

S3.3. Fourier-transform infrared spectroscopy (FTIR)

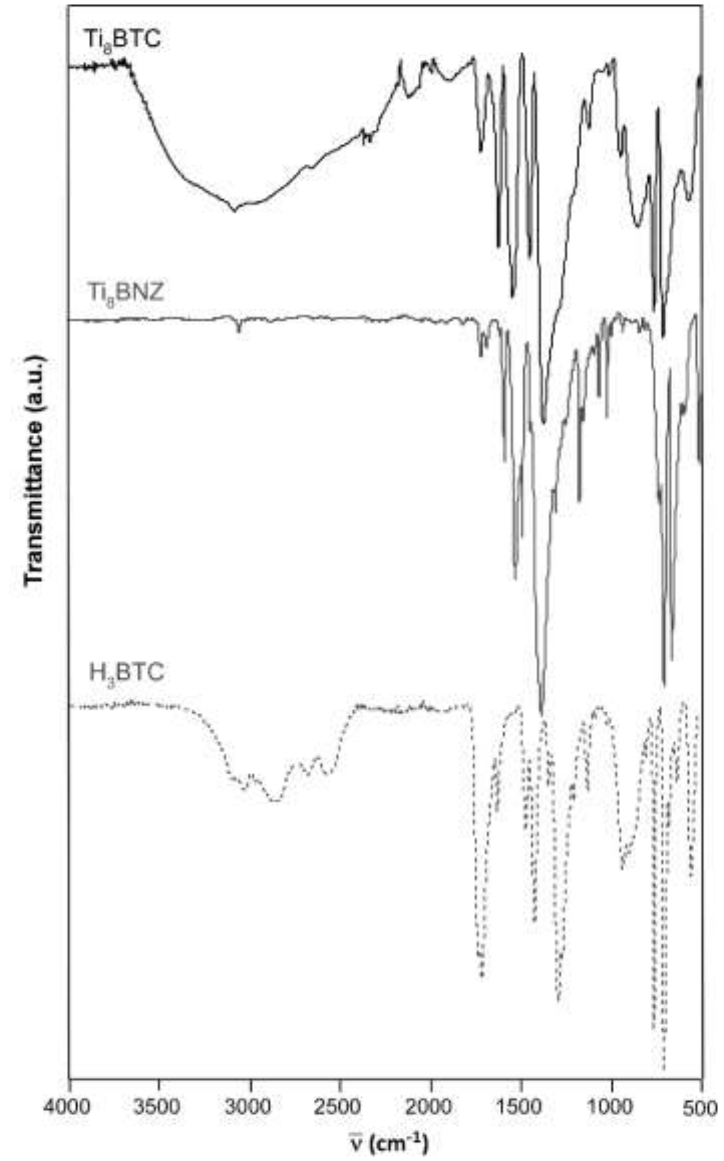

Fig. S7 FTIR spectra comparison of Ti_8BTC, Ti_8BNZ precursor and H_3BTC compound.

Table S3 Assignment of the signals of the FTIR spectra shown in Fig. S7.

VIBRATION TYPE	Ti_8BTC	Ti_8BNZ	H_3BTC
**ν (O-H)**	3410 m	-	3100 – 2900 s
**ν (C-H)**	3080, 2975, 2930 m	3070, 3025 w	3060, 2860 s
**ν (C=O)**	1705 m	-	1690 f
**$ν_{ring}$ (C=C)**	1610 s	1600, 1590 m	1605 m
**V_{asy} (COO)**	1540 m	1530 m	-
**$ν_{ring}$ (C=C)**	1440 m	1490 m	1450 m
**ν (C–O)**		-	1400 m
**V_{sym} (COO)**	1375 s	1380 s	
**$δ_{oop}$ (O-H)**			1325 w
**V (C–COO)**	1270 sh	1305 m	1260 s
**$δ_{ip}$ (C-H)**	1110 w	1175, 1155 m	1238 m, 1110 w
**ν (Ti-O)**	580, 450 w	585, 450 m	-

[a]: ν = stretching; δ = bending; ip = in-plane; oop = out-of-plane; asy = antisymmetric, sym = symmetric, s = strong, m = medium, w = weak; sh = shoulder.

Quantification of accesible –COOH groups:

The presence and accessibility of free carboxylic acid (–COOH) groups in Ti_8BTC were evaluated using FTIR spectroscopy combined with the cumulative standard addition method. This approach, although is not typical, allowed us to estimate the total and accessible amount of –COOH groups present in the material. The standard addition method allows to compensate the matrix effects that can affect the intensity of characteristic vibrational bands. The integrated area of the ν (C=O) stretching band, located at 1705 cm^{-1}, was used as the quantitative parameter.[10,11]

In the process, the total –COOH content in Ti_8BTC was initially determined by sequentially adding known small amounts of H_3BTC to the sample and recording the FTIR spectra after each addition. The first measurement corresponds to the pristine Ti_8BTC sample, whose –COOH content is unknown. Subsequently, incremental amounts of H_3BTC were added in successive samples, and the corresponding spectral changes were used to construct a standard addition curve (Fig. S8a). This procedure provides a linear relationship whose x-axis intercept corresponds to the intrinsic –COOH content initially present in the Ti_8BTC sample (before additions).

The obtained value (1.06 mmol·g^{-1} or *ca*. 2.0 mol of COOH per mol of MOA) was then used to construct an external calibration curve (Fig. S8b) for the ν(C=O) band area as a function of the amount of free –COOH groups present, and it was subsequently employed to quantify by extrapolation the remaining –COOH groups in Ti_8BTC after treatment with ammonia, which selectively neutralizes surface-accessible carboxylic sites by forming (NH_4^+)($COO^−$) pairs. This second determination corresponds to the non-accessible –COOH groups within the framework (0.75 mmol·g^{-1}). The difference between both measurements provides the surface-accessible –COOH fraction, *i.e.*, those available for coordination with external metal complexes during post-synthetic doping, resulting in 0.31 mmol·g^{-1} or *ca*. 0.6 mol of COOH per mol of MOA, which represents approximately the 30% of the total acid sites.

[10] G. Hutchinson, C. D. M. Welsh and J. Burés, Use of Standard Addition to Quantify In Situ FTIR Reaction Data. *J. Org. Chem*. 2021, **86** (2), 2012–2016.
[11] P. Zang, J. He and X. Zhou, An FTIR Standard Addition Method for Quantification of Bound Styrene in Its Copolymers. *Polym. Test.* 2008, **27** (2), 153–157.

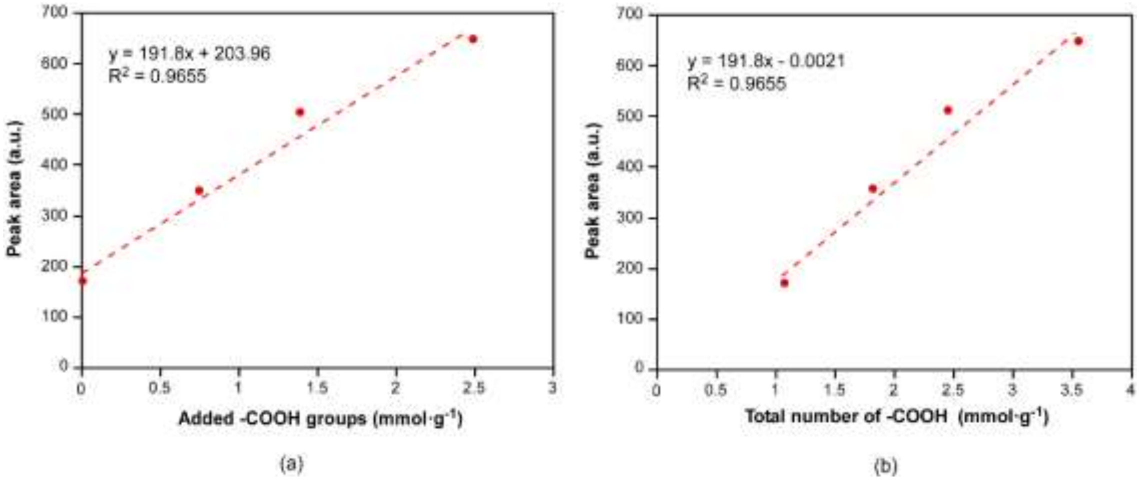

Fig. S8 (a) Standard additions and (b) external calibration curves used to estimate the total and accessible –COOH groups in T_8BTC, respectively.

S3.4. Powder X-ray diffraction (PXRD)

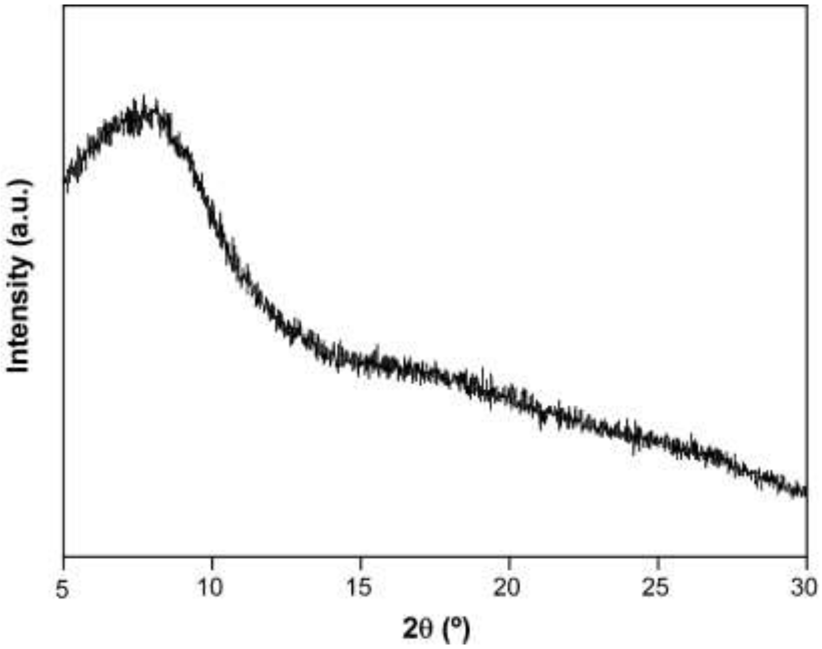

Fig. S9 PXRD pattern of Ti_8BTC aerogel.

S3.5. Transmission electron microscopy (TEM)

Additional HAADF-TEM images and elemental mapping analyses for CoTPY@Ti_8BTC, NiTPY@Ti_8BTC and CuTPY@Ti_8BTC samples are provided here. These images further confirm the homogeneous distribution of different dopant metals within the metal-organic network.

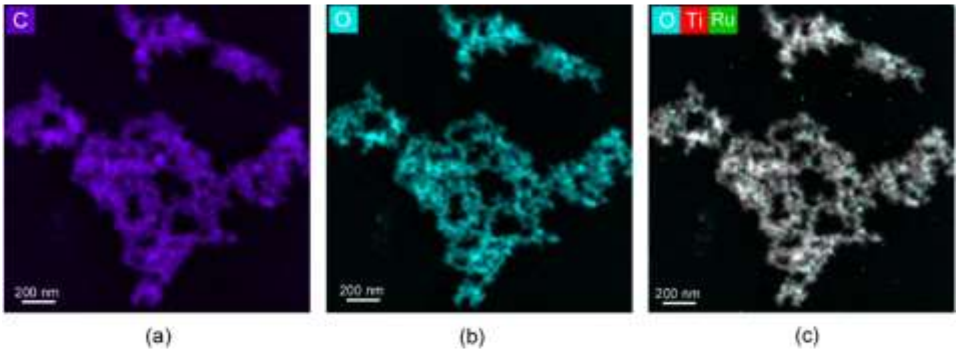

Fig. S10 Elemental mapping for (a) C, (b) O and (c) combined elemental mapping for O, Ti and Ru taken on RuTPY@Ti_8BTC aerogel.

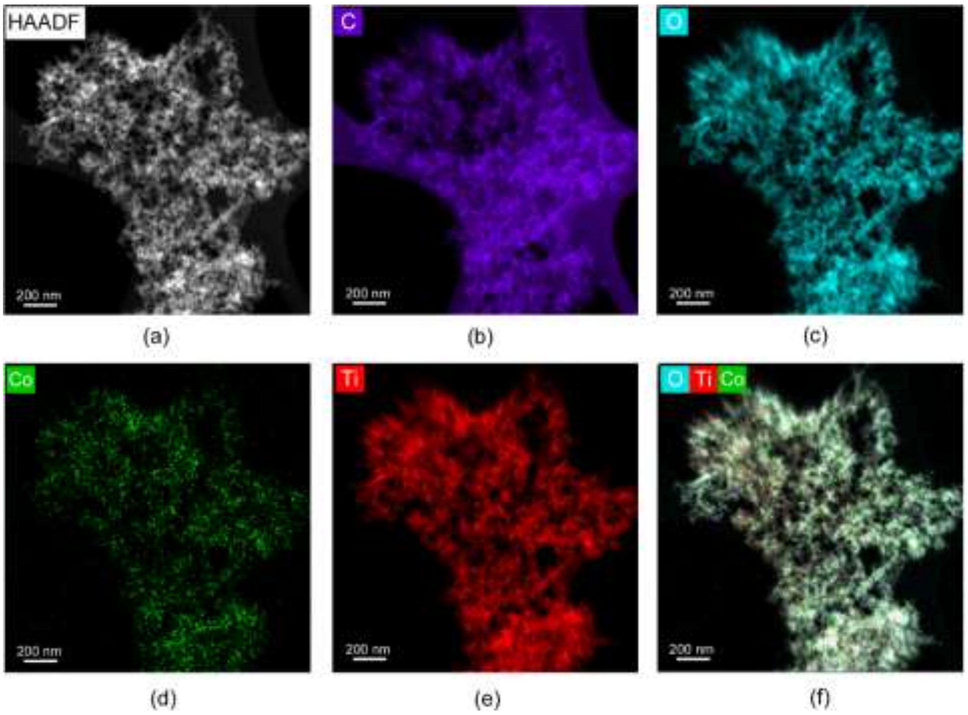

Fig. S11 (a) HAADF-TEM micrograph and elemental mapping for (b) C, (c) O, (d) Co, (e) Ti, and (f) combined elemental mapping for O, Ti and Co taken on CoTPY@Ti_8BTC aerogel. The carbon signal is primarily attributed to the TEM grid, but some material presence can still be observed.

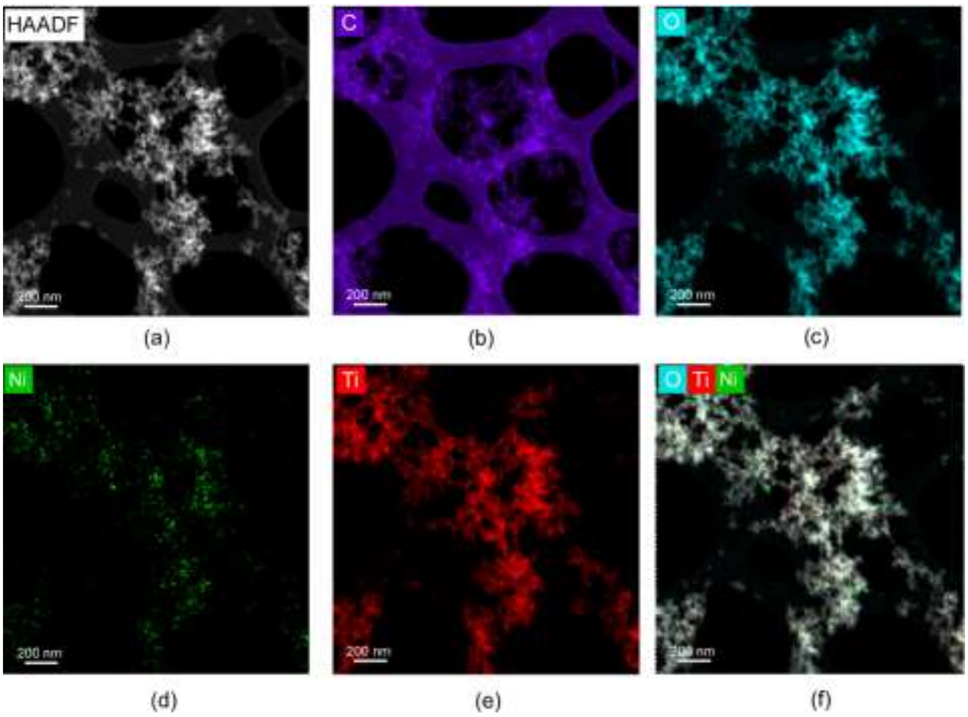

Fig. S12 (a) HAADF-TEM micrograph and elemental mapping for (b) C, (c) O, (d) Ni, (e) Ti, and (f) combined elemental mapping for O, Ti and Ni taken on NiTPY@Ti_8BTC aerogel. The carbon signal is primarily attributed to the TEM grid, but some material presence can still be observed.

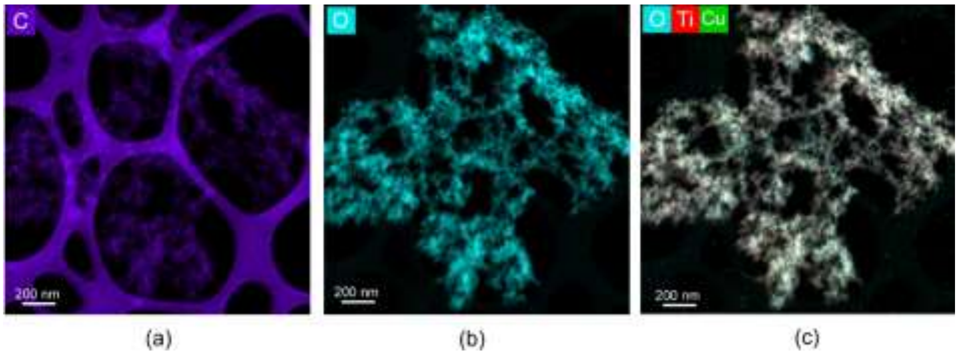

Fig. S13 Elemental mapping for (a) C, (b) O and (c) combined elemental mapping for O, Ti and Cu taken on CuTPY@Ti_8BTC aerogel.

S3.6. Scanning electron microscopy (SEM)

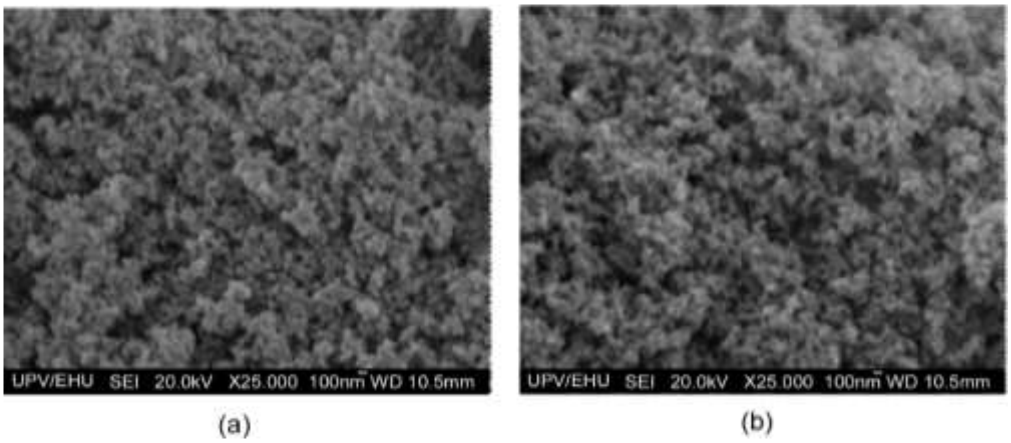

Fig. S14 SEM images (mode: SE) at 25 kX magnifications taken on (a) Ti_8BTC and (b) $RuCl_3$@Ti_8BTC aerogels.

S3.7. N_2 adsorption isotherms and cumulative pore size distribution

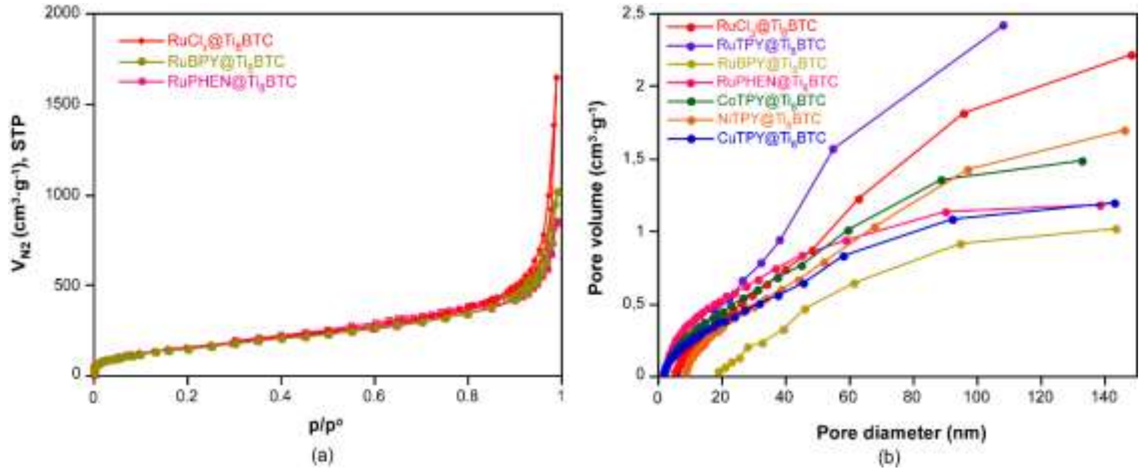

Fig. S15 (a) Nitrogen adsorption isotherm (77 K) for $RuCl_3@Ti_8BTC$, $RuBPY@Ti_8BTC$ and $RuPHEN@Ti_8BTC$. Closed symbols for adsorption curves and open symbols for desorption. (b) BJH cumulative pore size distribution for $RuCl_3@Ti_8BTC$, $RuTPY@Ti_8BTC$, $RuBPY@Ti_8BTC$, $RuPHEN@Ti_8BTC$, $CoTPY@Ti_8BTC$, $NiTPY@Ti_8BTC$ and $CuTPY@Ti_8BTC$.

S3.8. X-ray photoelectron spectroscopy (XPS)

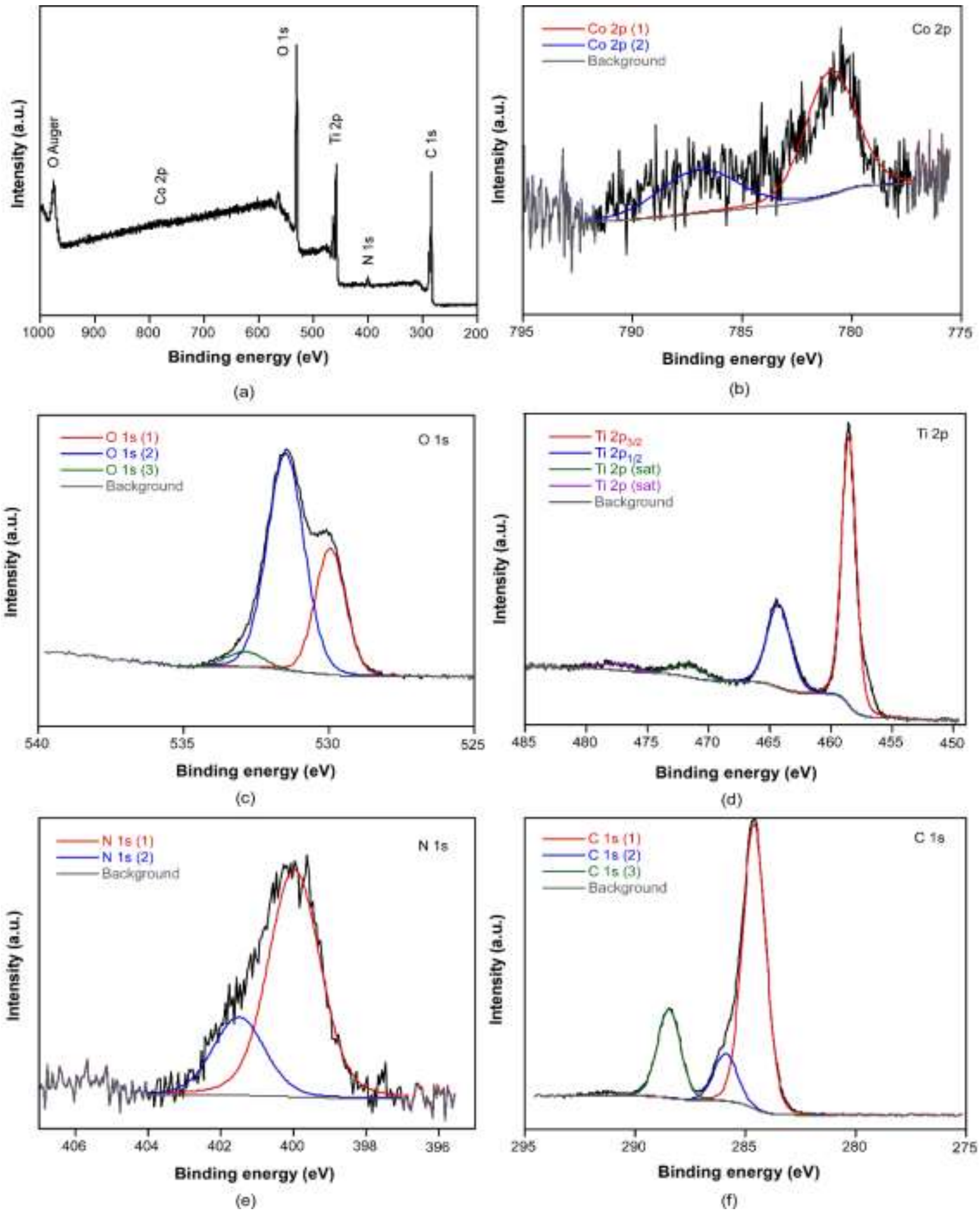

Fig. S16 (a) XPS full spectrum and high-resolution spectrum of (b) Co 2p, (c) O 1s, (d) Ti 2p, (e) N 1s, and (f) C 1s for CoTPY@Ti_8BTC. Envelopes are omitted for clarity.

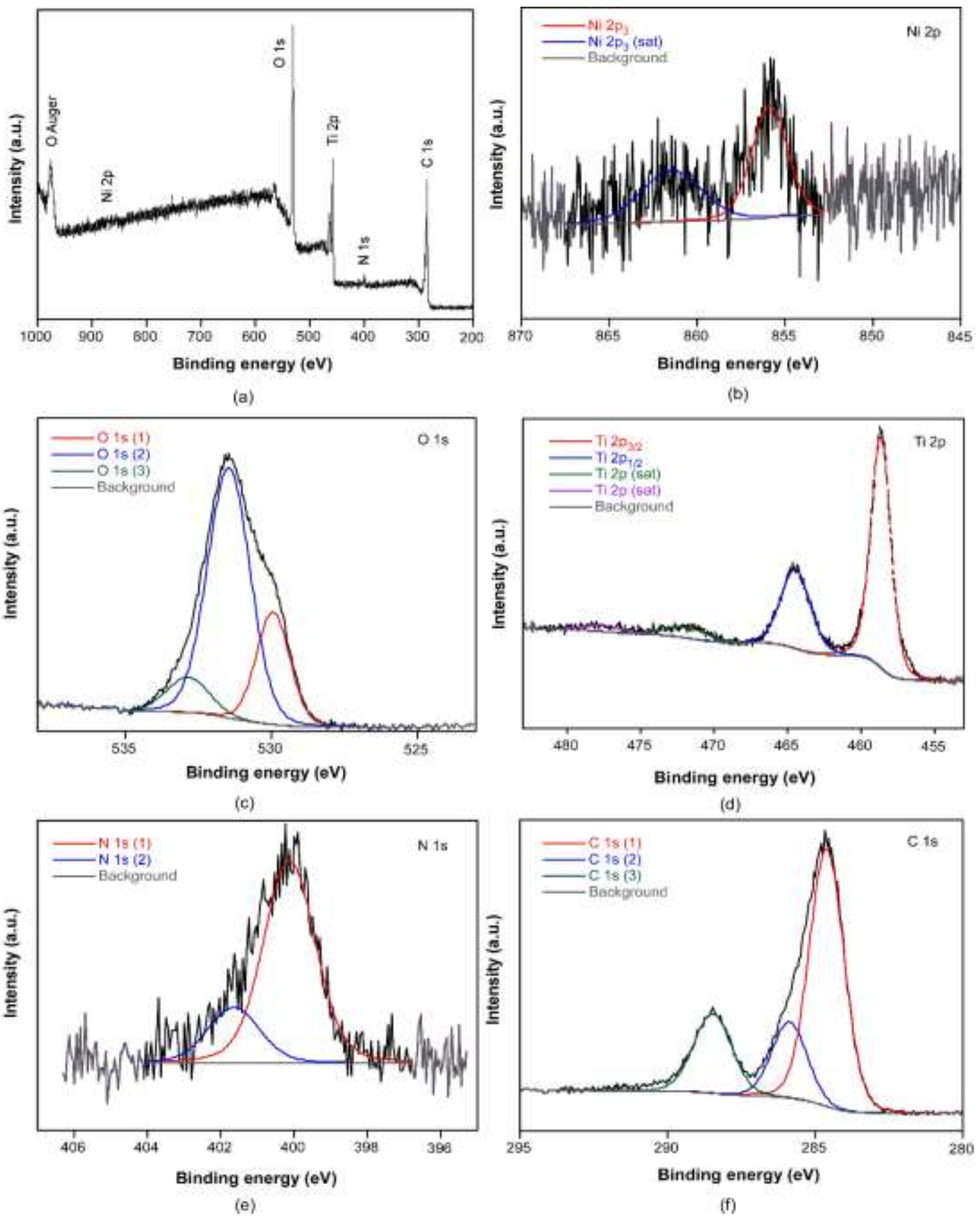

Fig. S17 (a) XPS full spectrum and high-resolution spectrum of (b) Ni 2p, (c) O 1s, (d) Ti 2p, (e) N 1s, and (f) C 1s for NiTPY@Ti_8BTC. Envelopes are omitted for clarity.

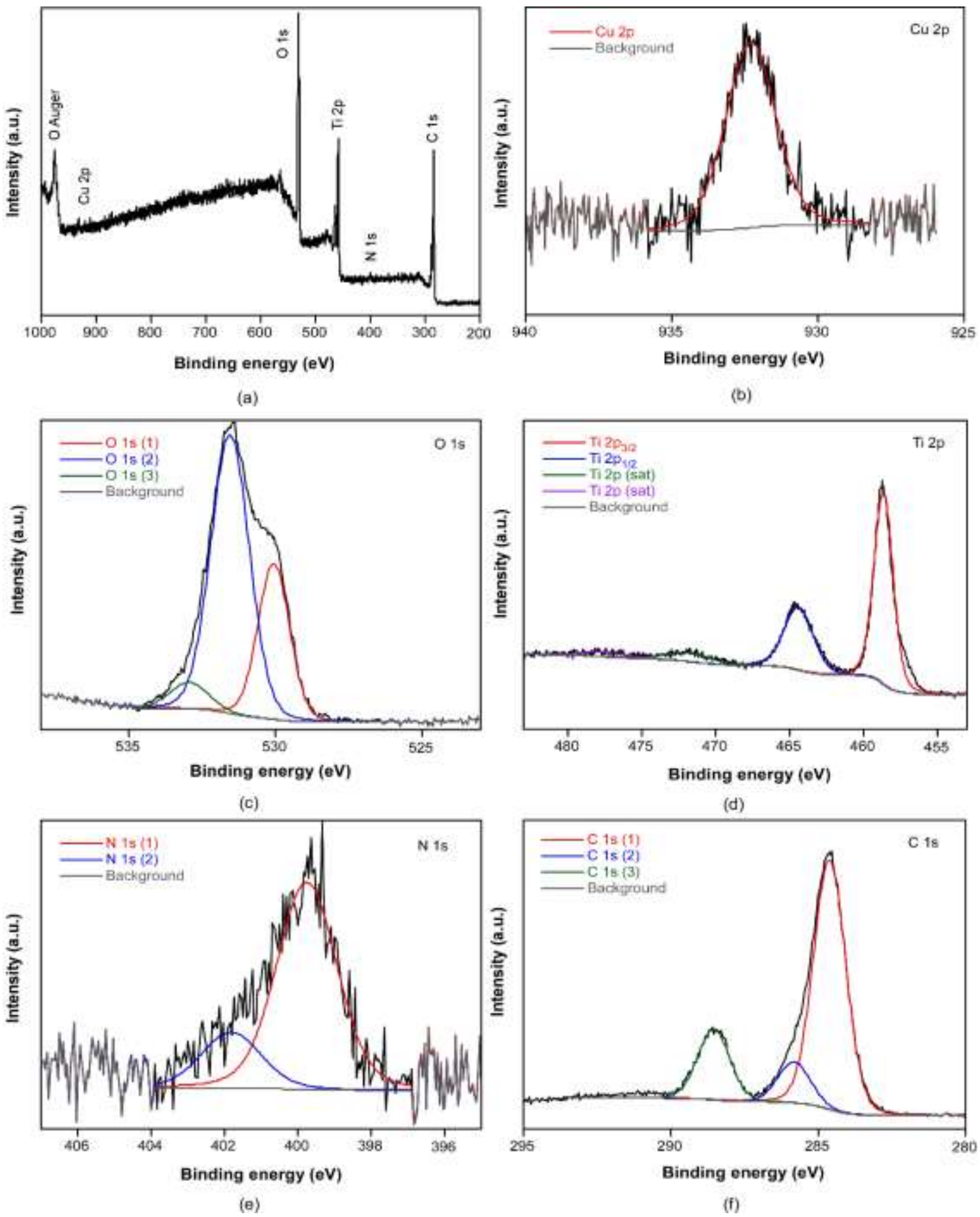

Fig. S18 (a) XPS full spectrum and high-resolution spectrum of (b) Cu 2p, (c) O 1s, (d) Ti 2p, (e) N 1s, and (f) C 1s for CuTPY@Ti_8BTC. Envelopes are omitted for clarity.

S3.9. UV-Vis diffuse reflectance spectroscopy (DRS)

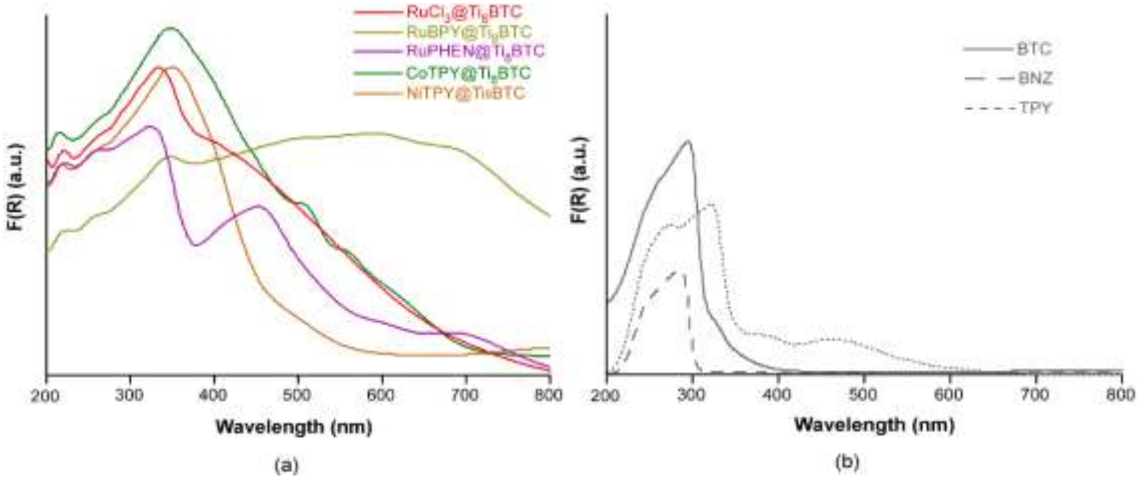

Fig. S19 UV–Vis absorbance spectra derived from the Kubelka–Munk function F(R) of (a) RuCl$_3$@Ti$_8$BTC, RuBPY@Ti$_8$BTC, RuPHEN@Ti$_8$BTC, CoTPY@Ti$_8$BTC and NiTPY@Ti$_8$BTC samples and (b) H$_3$BTC, HBNZ and TPY compounds.

<u>**Optical band gap calculations for Ti$_8$BTC**</u>

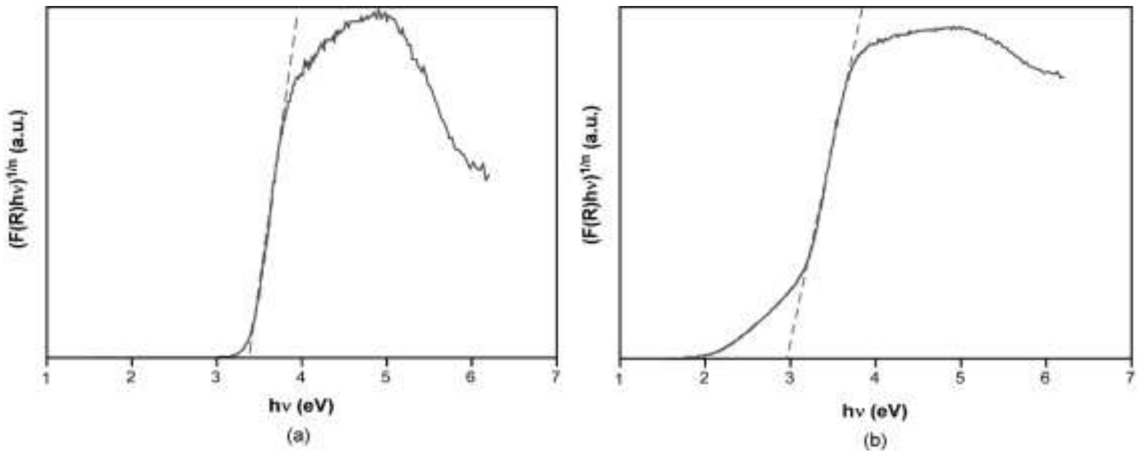

Fig. S20 (F(R)hν)$^{1/n}$ vs. hν plots for Ti$_8$BTC, depicting the linear fit to Tauc equation (dashed lines) for (a) direct with n = 0.5 and (b) indirect with n = 2 band gaps.

S3.10. Photoluminescence (PL) measurements

Photoluminescence (PL) spectra in fluorescence were recorded to assess the radiative recombination of photogenerated charge carriers in the Ti$_8$BTC-based materials. The organic linker H$_3$BTC exhibits a broad emission band centred at 430 nm when excited at 365 nm. Upon coordination with titanium to form Ti$_8$BTC, this emission is almost completely quenched, indicating an efficient suppression of radiative deactivation pathways (Fig. S21). The RuTPY@Ti$_8$BTC and CuTPY@Ti$_8$BTC samples also show no detectable PL signals under the same excitation conditions, consistent again with strong non-radiative deactivation of excited states. This behaviour is attributed to the presence of transition-metal centres with partially filled *d* orbitals, which can promote ligand-to-metal charge transfer (LMCT) processes that compete with fluorescence emission.

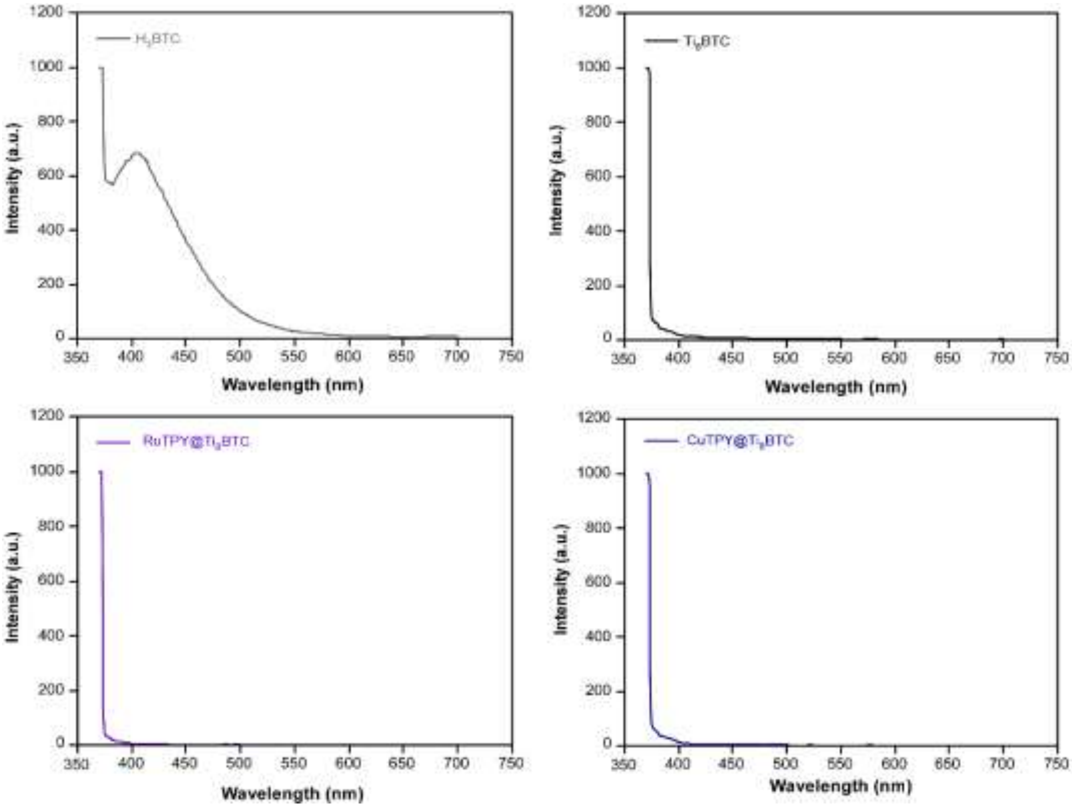

Fig. S21 Fluorescence spectra of H_3BTC, Ti_8BTC, RuTPY@Ti_8BTC, and CuTPY@Ti_8BTC recorded under 365 nm excitation wavelength.

S4. PHOTOCATALYTIC HYDROGEN PRODUCTION

S4.1. Hydrogen evolution experiments

Table S4 HER activity and associated errors for the experiments performed to optimize the distance between the light source and the reactor. Conditions: 10 mg catalyst in 8 mL of 0.1 M TEOA aq. solution (pH 7) using UV light source after 2 h of reaction.

Distances (cm)	H_2 production ($\mu mol \cdot h^{-1} \cdot g^{-1}$)
**7**	49.43 ± 1.62
**8**	85.26 ± 4.48
**9**	42.89 ± 0.15
**10**	39.20 ± 1.48

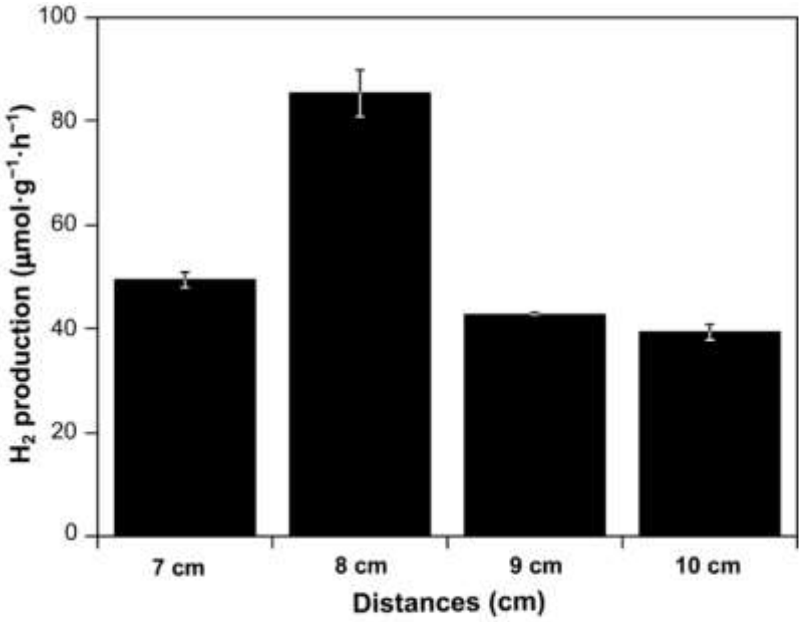

Fig. S22 Influence of the distance between the light source and the reactor in HER activity provided by Ti_8BTC. Conditions: 10 mg catalyst in 8 mL of 0.1 M TEOA aq. solution (pH 7) using UV light source after 2 h of reaction.

Table S5 Hydrogen production rate and HER activity with associated errors for experiments performed varying Ti_8BTC concentrations (C_1: 625 mg·L^{-1}; C_2: 1250 mg·L^{-1}; C_3: 2500 mg·L^{-1}) after 2 h of reaction. Conditions: 5, 10 and 20 mg, respectively, in 8 mL of 0.1 M TEOA aq. solution (pH 7) at 8 cm from UV light source.

	Ti_8BTC (625 mg·L^{-1})	Ti_8BTC (1250 mg·L^{-1})	Ti_8BTC (2500 mg·L^{-1})
**Rate ($\mu mol \cdot h^{-1}$)**	72.94 ± 4.07	113.81 ± 12.32	174.42 ± 25.02
**Activity ($\mu mol \cdot g^{-1} \cdot h^{-1}$)**	110.87 ± 0.83	85.26 ± 4.48	67.29 ± 8.88

Table S6 HER activity and associated error values for experiments performed with Ti_8BTC doped with metal complexes of terpyridine (TPY) after 2h of reaction. Conditions: 5 mg of catalyst in 8 mL (C = 625 mg·L^{-1}) of 0.1 M TEOA aq. solution (pH 7) at 8 cm from UV light source.

TPY metal complexes	H_2 production ($\mu mol \cdot g^{-1} \cdot h^{-1}$)
**Ti_8BTC**	110.87 ± 0.83
**RuTPY@Ti_8BTC**	167.05 ± 7.53
**CoTPY@Ti_8BTC**	105.50 ± 6.73
**NiTPY@Ti_8BTC**	83.05 ± 3.31
**CuTPY@Ti_8BTC**	163.73 ± 20.19

Table S7 HER activity during 24 h of reaction for Ti_8BTC, ~~and~~ RuTPY@Ti_8BTC and CuTPY@Ti_8BTC with corresponding associated errors. Conditions: 5 mg of catalyst in 8 mL (C = 625 mg·L^{-1}) of 0.1 M TEOA aq. solution (pH 7) at 8 cm from UV light source.

**Time (min)**	**Ti_8BTC H_2 production (μmol·g^{-1}·h^{-1})**	**RuTPY@Ti_8BTC H_2 production (μmol·g^{-1}·h^{-1})**	**CuTPY@Ti_8BTC H_2 production (μmol·g^{-1}·h^{-1})**
**0**	-	-	-
**15**	-	-	-
**30**	-	17.19 ± 24.31	-
**45**	28.57 ± 40.40	57.07 ± 6.55	50.11 ± 4.01
**60**	61.26 ± 32.46	136.63 ± 0.70	118.95 ± 5.28
**75**	74.68 ± 23.23	165.76 ± 3.50	149.25 ± 7.71
**90**	86.76 ± 14.72	177.78 ± 3.64	163.94 ± 8.88
**105**	97.42 ± 12.13	177.38 ± 2.21	165.03 ± 11.09
**120**	99.44 ± 1.08	179.18 ± 2.29	169.66 ± 7.22
**135**	101.15 ± 3.55	175.45 ± 2.13	166.52 ± 9.09
**150**	108.58 ± 0.89	177.20 ± 7.55	167.03 ± 0.66
**165**	104.93 ± 4.69	176.45 ± 2.17	164.40 ± 6.80
**180**	105.76 ± 3.25	175.95 ± 2.15	159.82 ± 10.67
**195**	104.55 ± 3.83	178.43 ± 2.26	161.01 ± 4.41
**210**	107.74 ± 1.06	174.43 ± 2.09	163.04 ± 8.81
**225**	105.65 ± 4.03	175.36 ± 2.13	162.70 ± 6.67
**240**	108.89 ± 2.04	174.69 ± 2.10	161.89 ± 8.51
**255**	107.45 ± 0.53	175.17 ± 2.12	163.71 ± 4.63
**270**	105.51 ± 0.73	173.96 ± 2.07	158.46 ± 8.45
**285**	106.10 ± 0.58	175.45 ± 2.13	157.23 ± 8.35
**300**	105.74 ± 0.53	170.71 ± 1.93	161.43 ± 8.68
**315**	104.85 ± 0.57	173.15 ± 2.03	159.49 ± 8.53
**330**	103.65 ± 0.06	169.88 ± 1.89	156.08 ± 6.82
**345**	104.97 ± 1.29	170.27 ± 1.91	155.65 ± 5.98
**360**	103.44 ± 1.00	170.64 ± 1.93	154.78 ± 11.95
**375**	102.52 ± 4.47	168.86 ± 1.85	151.26 ± 8.89
**390**	103.54 ± 1.38	168.69 ± 1.84	149.97 ± 8.55
**405**	100.56 ± 2.73	163.41 ± 0.16	149.13 ± 9.87
**420**	102.45 ± 5.72	166.82 ± 1.76	150.93 ± 7.21
**435**	103.31 ± 3.44	163.84 ± 1.64	149.05 ± 11.20
**450**	101.54 ± 4.89	166.50 ± 1.36	146.49 ± 7.39
**465**	102.07 ± 4.03	165.94 ± 1.63	144.84 ± 9.93
**480**	100.66 ± 6.28	164.69 ± 3.60	142.44 ± 7.88
**495**	100.78 ± 6.02	163.17 ± 1.77	143.13 ± 5.37
**510**	101.92 ± 8.36	161.65 ± 3.58	144.90 ± 6.79
**525**	102.13 ± 9.12	164.11 ± 1.22	146.04 ± 6.39
**540**	102.18 ± 8.48	165.35 ± 2.93	143.15 ± 1.94
**555**	102.27 ± 10.55	164.45 ± 1.67	149.27 ± 4.48
**570**	102.06 ± 9.20	163.90 ± 2.42	149.18 ± 11.46
**585**	101.19 ± 9.40	164.64 ± 0.23	147.90 ± 5.31
**600**	101.90 ± 7.29	165.92 ± 1.87	150.16 ± 6.98
**615**	101.04 ± 6.25	165.07 ± 2.77	151.39 ± 6.94
**630**	101.80 ± 8.30	163.43 ± 2.86	149.23 ± 7.51
**645**	100.48 ± 5.85	163.67 ± 0.03	144.75 ± 9.77
**660**	102.58 ± 4.60	162.17 ± 5.51	147.29 ± 5.53
**675**	101.24 ± 4.30	161.91 ± 0.95	147.08 ± 10.12
**690**	101.64 ± 5.67	161.39 ± 2.94	143.44 ± 9.78
**705**	102.05 ± 6.04	159.65 ± 2.49	144.27 ± 6.36
**720**	102.18 ± 4.27	157.90 ± 2.84	146.59 ± 7.36
**735**	103.48 ± 5.09	155.57 ± 1.96	146.77 ± 7.40
**750**	102.09 ± 3.66	156.11 ± 2.37	145.82 ± 8.97
**765**	103.80 ± 5.73	155.63 ± 0.48	144.19 ± 8.03
**780**	101.09 ± 8.96	158.10 ± 0.41	145.48 ± 6.45
**795**	102.42 ± 6.58	156.15 ± 4.46	144.46 ± 9.93
**810**	103.16 ± 4.97	159.82 ± 3.07	145.47 ± 2.98
**825**	103.26 ± 10.27	155.76 ± 1.58	144.93 ± 8.63
**840**	102.12 ± 5.58	156.86 ± 3.79	143.49 ± 7.97
**855**	104.80 ± 7.42	155.43 ± 4.51	143.44 ± 6.52
**870**	103.55 ± 4.52	155.19 ± 4.16	144.77 ± 7.52
**885**	103.78 ± 5.54	154.09 ± 2.80	143.89 ± 6.90
**900**	102.37 ± 5.72	155.63 ± 0.64	144.16 ± 7.66
**915**	103.59 ± 5.68	154.23 ± 2.39	142.69 ± 8.98
**930**	104.17 ± 7.28	156.29 ± 4.09	140.25 ± 5.92
**945**	103.77 ± 7.23	154.43 ± 2.77	141.76 ± 6.04
**960**	104.61 ± 7.35	155.56 ± 2.46	141.65 ± 3.53
**975**	103.66 ± 7.84	154.66 ± 2.33	143.01 ± 9.19

**990**	105.49 ± 9.83	153.09 ± 6.58	142.42 ± 5.83
**1005**	105.55 ± 9.69	154.93 ± 2.77	142.80 ± 8.12
**1020**	105.91 ± 8.08	152.13 ± 0.15	140.18 ± 9.86
**1035**	104.66 ± 6.67	153.95 ± 4.09	140.02 ± 7.90
**1050**	105.30 ± 9.06	155.58 ± 0.69	140.20 ± 5.68
**1065**	104.52 ± 6.46	155.44 ± 0.89	140.96 ± 7.25
**1080**	101.92 ± 5.10	157.86 ± 1.75	141.60 ± 5.90
**1095**	104.25 ± 8.71	156.38 ± 2.26	140.47 ± 10.05
**1110**	103.39 ± 6.42	157.70 ± 1.21	139.20 ± 9.30
**1125**	103.70 ± 6.32	153.94 ± 3.76	137.66 ± 10.31
**1140**	105.07 ± 9.91	157.00 ± 2.54	139.23 ± 9.03
**1155**	106.36 ± 8.69	155.70 ± 2.44	138.13 ± 10.00
**1170**	104.46 ± 8.89	154.77 ± 3.24	140.92 ± 5.03
**1185**	103.55 ± 6.72	154.67 ± 1.51	140.85 ± 9.39
**1200**	106.79 ± 9.12	154.96 ± 1.14	142.25 ± 6.90
**1215**	106.30 ± 9.35	155.27 ± 0.12	142.94 ± 6.46
**1230**	103.98 ± 7.04	155.54 ± 2.99	141.87 ± 9.54
**1245**	103.36 ± 8.76	154.81 ± 3.14	140.78 ± 5.94
**1260**	103.67 ± 8.54	153.27 ± 2.67	142.55 ± 5.75
**1275**	104.57 ± 9.82	152.37 ± 4.54	140.79 ± 6.77
**1290**	104.86 ± 9.68	153.87 ± 0.02	141.67 ± 10.57
**1305**	103.14 ± 6.89	151.73 ± 2.34	139.09 ± 11.36
**1320**	103.17 ± 7.96	154.30 ± 1.90	141.00 ± 7.04
**1335**	101.95 ± 7.75	151.23 ± 0.52	140.58 ± 7.65
**1350**	101.49 ± 6.18	153.05 ± 2.29	138.82 ± 8.79
**1365**	101.44 ± 6.69	151.59 ± 1.21	138.89 ± 8.55
**1380**	101.24 ± 6.90	153.41 ± 0.51	139.40 ± 8.20
**1395**	100.35 ± 7.57	152.26 ± 0.06	140.36 ± 8.05
**1410**	101.56 ± 7.32	152.80 ± 3.87	138.71 ± 8.15
**1425**	101.45 ± 6.61	152.03 ± 2.62	138.54 ± 5.66
**1440**	101.79 ± 7.60	151.04 ± 0.79	140.20 ± 5.83
**1455**	101.10 ± 6.05	151.63 ± 0.12	141.71 ± 6.14
**1470**	99.76 ± 7.19	150.22 ± 2.57	136.90 ± 9.18
**1485**	100.54 ± 5.17	149.58 ± 5.22	141.34 ± 6.80
**1500**	100.53 ± 8.40	150.26 ± 3.43	139.58 ± 8.84

S4.2. Apparent quantum efficiency (AQE) calculations

To quantify the efficiency of the photocatalytic process and normalize the HER activity across samples, the apparent quantum efficiency (AQE) was calculated at 365 nm for Ti_8BTC, RuTPY@Ti_8BTC, and CuTPY@Ti_8BTC. The calculations were based on the incident optical power (112 mW), the irradiation time, and the amount of evolved hydrogen. The obtained AQE values were 0.091% (Ti_8BTC), 0.147% (RuTPY@Ti_8BTC), and 0.137% (CuTPY@Ti_8BTC). These results confirm that the enhanced hydrogen-evolution performance of the Ru- and Cu-functionalized materials cannot be solely explained by differences in photon flux.

The following constants and experimental parameters were used in the calculations:

- Reported activity: 110 (Ti_8BTC), 167 (RuTPY@Ti_8BTC), and 164 (CuTPY@Ti_8BTC) $\mu mol \cdot g^{-1} \cdot h^{-1}$
- Catalyst mass used: ~ 5 mg
- Irradiation time: 2 h = 7200 s
- Incident power at 365 nm: $P = 0.112\ J \cdot s^{-1}$
- Constants: $h = 6.626 \cdot 10^{-34}\ J \cdot s$, $c = 2.998 \cdot 10^{8}\ m \cdot s^{-1}$, $N_A = 6.022 \cdot 10^{23}\ mol^{-1}$

The AQE was calculated from the amount of hydrogen produced, the number of electrons transferred, and the number of incident photons, using the following equations:

$$AQE(\%) = \frac{n_{electrons}}{n_{photons}} \cdot 100;\ N_{electrons} = 2 \cdot n_{H_2} \cdot N_A;\ N_{photons} = \frac{P \cdot t}{E_{photon}} = \frac{P \cdot t}{\frac{hc}{\lambda}}$$
